# RIPK1 protects naive and regulatory T cells from TNFR1-induced apoptosis

**DOI:** 10.1038/s41418-024-01301-w

**Published:** 2024-05-11

**Authors:** Jelle Huysentruyt, Wolf Steels, Mario Ruiz Perez, Bruno Verstraeten, Mike Vadi, Tatyana Divert, Kayleigh Flies, Nozomi Takahashi, Bart N. Lambrecht, Wim Declercq, Tom Vanden Berghe, Jonathan Maelfait, Peter Vandenabeele, Peter Tougaard

**Affiliations:** 1 Cell death and Inflammation Unit, VIB-UGent Center for Inflammation Research, Ghent, Belgium; 2 Department of Biomedical Molecular Biology, Ghent University, Ghent, Belgium; 3 Department of Internal Medicine and Pediatrics, Faculty of Medicine and Health Sciences, Ghent University, Ghent, Belgium; 4 Laboratory of Mucosal Immunology, VIB-UGent Center for Inflammation Research, Ghent University, Ghent, Belgium; 5 Department of Respiratory Medicine, Ghent University Hospital, Ghent, Belgium; 6 Department of Pulmonary Medicine, Erasmus MC, Rotterdam, Netherlands; 7 Department of Biomedical Sciences, University of Antwerp, Antwerp, Belgium

**Keywords:** Immunology, Cell biology

## Abstract

The T cell population size is stringently controlled before, during, and after immune responses, as improper cell death regulation can result in autoimmunity and immunodeficiency. RIPK1 is an important regulator of peripheral T cell survival and homeostasis. However, whether different peripheral T cell subsets show a differential requirement for RIPK1 and which programmed cell death pathway they engage in vivo remains unclear. In this study, we demonstrate that conditional ablation of *Ripk1* in conventional T cells (*Ripk1*^ΔCD4^) causes peripheral T cell lymphopenia, as witnessed by a profound loss of naive CD4^+^, naive CD8^+^, and FoxP3^+^ regulatory T cells. Interestingly, peripheral naive CD8^+^ T cells in *Ripk1*^ΔCD4^ mice appear to undergo a selective pressure to retain RIPK1 expression following activation. Mixed bone marrow chimeras revealed a competitive survival disadvantage for naive, effector, and memory T cells lacking RIPK1. Additionally, tamoxifen-induced deletion of RIPK1 in CD4-expressing cells in adult life confirmed the importance of RIPK1 in post-thymic survival of CD4^+^ T cells. *Ripk1*^K45A^ mice showed no change in peripheral T cell subsets, demonstrating that the T cell lymphopenia was due to the scaffold function of RIPK1 rather than to its kinase activity. Enhanced numbers of *Ripk1*^ΔCD4^ naive T cells expressed the proliferation marker Ki-67^+^ despite the peripheral lymphopenia and single-cell RNA sequencing revealed T cell-specific transcriptomic alterations that were reverted by additional caspase-8 deficiency. Furthermore, *Ripk1*^ΔCD4^*Casp8* ^ΔCD4^ and *Ripk1*^ΔCD4^*Tnfr1*^−/−^ double-knockout mice rescued the peripheral T cell lymphopenia, revealing that RIPK1-deficient naive CD4^+^ and CD8^+^ cells and FoxP3^+^ regulatory T cells specifically die from TNF- and caspase-8-mediated apoptosis in vivo. Altogether, our findings emphasize the essential role of RIPK1 as a scaffold in maintaining the peripheral T cell compartment and preventing TNFR1-induced apoptosis.

## Introduction

Conventional T lymphocytes are considered naive until encountering their cognate antigen in the periphery, after which they differentiate into effector and memory T cells. T cell homeostasis is strictly regulated before, during, and after infection to maintain the population size and establish optimal responsiveness [1, 2]. The need for stringent regulation of cell death in T lymphocytes has been underscored by the discovery of mutations in apoptosis mediators resulting in defective immune responses. Deficiencies in the death receptor-mediated pathway of extrinsic apoptosis, such as mutations in Fas or FasL, have been associated with autoimmune lymphoproliferative syndrome (ALPS) in both humans [3, 4] and mice [5, 6] due to inefficient clearing of antigen-activated T cells. Downstream of death receptors, T cell-specific deletion of FADD or caspase-8 in young mice resulted in immunodeficiency due to proliferation defects rather than lymphoproliferative-like syndrome [7, 8]. This was later explained by an increased sensitivity of FADD- or caspase-8-deficient peripheral T cells to necroptosis [9, 10].

Receptor-Interacting Protein Kinase 1 (RIPK1) is a central mediator of inflammation and cell death downstream of TNF-receptor 1 (TNFR1). By default, RIPK1 acts as a scaffold for the recruitment of signaling partners involved in TNF-mediated NF-κB and MAPK activation and subsequent induction of genes involved in cell survival and inflammation [11]. Several kinase-mediated checkpoints keep RIPK1 in pro-survival mode and negatively affect kinase-dependent cell death [12]. RIPK1 becomes an active kinase when these checkpoints are bypassed and induces caspase-8-dependent apoptosis [13, 14]. Furthermore, upon caspase-8 inhibition, RIPK1 can initiate the execution of necroptosis [15]. Biallelic *Ripk1* mutations in humans are associated with T cell dysbiosis and autoinflammatory syndromes [16–18]. Patients carrying *Ripk1* mutations rendering RIPK1 resistant to caspase-8 cleavage develop an autoinflammatory syndrome [19, 20]. Contrary to humans, whole-body RIPK1-deficient mice display postnatal lethality [21]. RIPK1-deficient fetal liver hematopoietic cells failed to reconstitute the peripheral T cell compartment in irradiated recipient mice [22]. Additionally, hematopoietic reconstitution experiments have shown that the reduction in RIPK1 deficient leukocyte populations in the bone marrow is restored by the administration of anti-TNF antibodies [23].

T cell-specific deletion of *Ripk1* induced peripheral T cell lymphopenia [24, 25]. Moreover, loss of RIPK1 in T cells apparently results in a senescent phenotype associated with age-related features such as neurodegeneration, sarcopenia, and inflammation in the lungs, kidneys, and heart [25]. In vivo ablation of *Ripk1* had no noticeable effect on the development of conventional TCRαβ^+^ T cell precursors in the thymus [26]. However, the effect of *Ripk1*-deficiency on peripheral T cell homeostasis remains unclear. Altogether, our data reveal that the RIPK1 scaffold function is required to protect peripheral naive and regulatory T cells from TNFR1- and Caspase-8-mediated apoptosis in vivo.

## Results

### RIPK1-deficiency in conventional T cells results in a vast loss of peripheral naive T cells and FoxP3^+^ Tregs

**Fig. 1:**
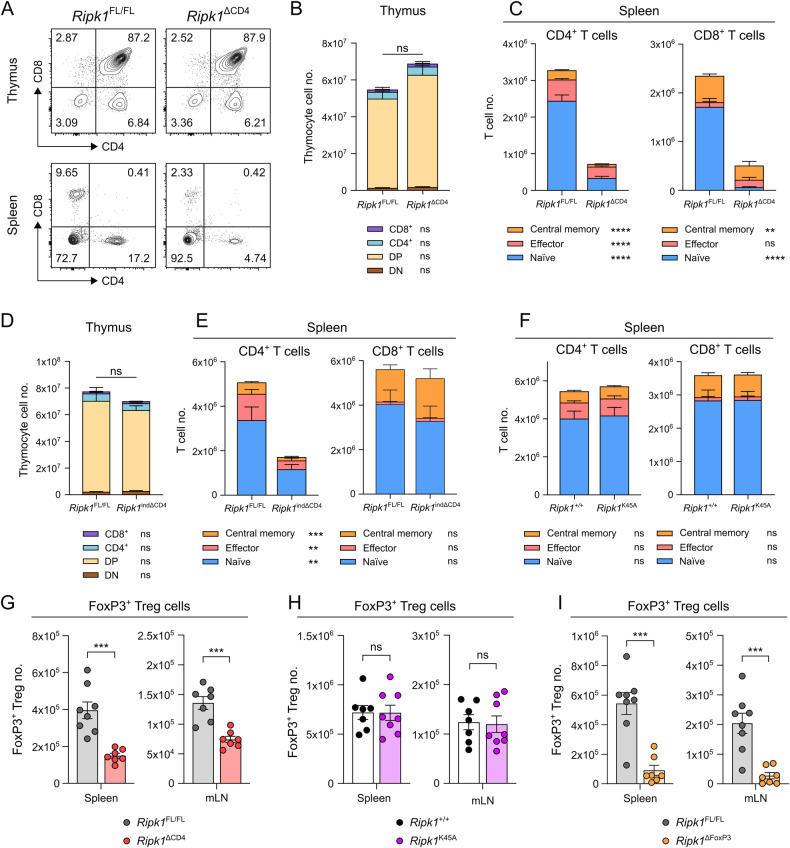
RIPK1-deficiency in conventional T cells results in a vast loss of peripheral naive T cells and FoxP3^+^ Tregs. **A** Flow cytometry plots of CD4 and CD8 expression in the thymus (upper) and spleen (lower) of *Ripk1*^ΔCD4^ mice and *Ripk1*^FL/FL^ littermates representative of at least three independent experiments. Percentage are displayed of each quadrant. **B** Stacked bar plots showing CD4^-^CD8^-^ double negative (DN), CD4^+^CD8^+^ double positive (DP), CD4^+^CD8^-^ single positive (CD4^+^ SP) and CD4^-^CD8^+^ single positive (CD8^+^ SP) cells in the thymus from (*n* = 11) *Ripk1*^ΔCD4^ mice and (*n* = 13) *Ripk1*^FL/FL^ littermates. **C** Stacked bar plots showing conventional CD4^+^ (left) and CD8^+^ (right) T cells, divided into naive (CD62L^+^CD44^low^), effector (CD62L^-^CD44^high^) and central memory (CD62L^+^CD44^high^) T cells in the spleen of (*n* = 7) *Ripk1*^ΔCD4^ mice and (*n* = 8) *Ripk1*^FL/FL^ littermates. *Ripk1*^indΔCD4^ mice and *Ripk1*^FL/FL^ littermates were treated with tamoxifen-containing food for ten weeks. Stacked bar plots showing (**D**) DN, DP, CD4^+^ SP, and CD8^+^ SP cells in the thymus from *n* = 3 mice per condition and (**E**) naive, effector, and central memory T cells within CD4^+^ (left) and CD8^+^ (right) populations in the spleen from (n = 3) *Ripk1*^indΔCD4^ mice and (*n* = 4) *Ripk1*^FL/FL^ littermates. **D**, **E** are representative of three independent experiments. **F** Stacked bar plots showing naive, effector, and central memory T cells within CD4^+^ (left) and CD8^+^ (right) populations in the spleen from (*n* = 8) *Ripk1*^ΔK45A^ mice and (*n* = 7) *Ripk1*^+/+^ littermates. Bar plots showing FoxP3^+^ regulatory T cells (Tregs) in the spleen (left) and mLN (right), (**G**) from (*n* = 7) *Ripk1*^ΔCD4^ mice and (*n* = 8) *Ripk1*^FL/FL^ littermates and (**H**) from (*n* = 8) *Ripk1*^ΔK45A^ mice and (*n* = 7) *Ripk1*^+/+^ littermates, and (**I**) from (*n* = 7) *Ripk1*^ΔFoxP3^ mice and (*n* = 8) *Ripk1*^FL/FL^ littermates. **I** Data are representative of three independent experiments, or (**C**, **F**, **G**, **H**) combined from two, or (**B**) three independent experiments. **B**–**I** Flow cytometry data are shown as mean ± sem, and (**G**–**I**) each dot represents an individual mouse. *Statistics*: statistical significance was calculated by (**G**–**I**) unpaired *t*-tests (two-sided) or (**B**–**F**) Fisher’s LSD two-way ANOVA on Log_2_-transformed data. ns non-significant, **p < 0.01, ***p < 0.001, ****p < 0.0001.

To investigate the role of RIPK1 in peripheral T lymphocytes, *Ripk1*^*fl/fl*^ and *Cd4-Cre* mice were crossed to generate mice with a specific deletion of *Ripk1* in conventional T cells (*Ripk1*^*ΔCD4*^). Similar to earlier observations [24, 26], we found no differences in total cell numbers or frequencies of immature CD4^−^CD8^−^ double-negative (DN) and CD4^+^CD8^+^ double positive DP thymocytes or mature CD4^+^ single-positive (SP) and CD8^+^ SP thymocytes (Figs. 1A, B, S1A). In contrast, the frequencies of CD4^+^ and CD8^+^ peripheral T cells were dramatically decreased in the spleen and mesenteric lymph nodes (mLN) of *Ripk1*^*ΔCD4*^ mice (Figs. 1A and S1B). Effector (CD62L^-^CD44^high^) and central memory (CD62L^+^CD44^high^) CD4^+^ and CD8^+^ T cells were reduced in the spleen and mLN, the decreased T cell numbers were primarily due to a loss of naive CD4^+^ and CD8^+^ T cells (CD62L^+^CD44^low^) (Figs. 1C and S1C).

*Ripk1*^*ΔCD4*^ mice initially develop normally, while older animals displayed reduced body weight compared to *Ripk1*^*fl/fl*^ littermates (Fig. S1D), as previously reported [25]. This report was performed on young adult mice (around 8–12 weeks-old) before any weight differences were observed (Fig. S1D). To determine whether the ablation of RIPK1 in fully developed mice would affect peripheral T cell survival in the same way as constitutive RIPK1 deletion in *Ripk1*^*ΔCD4*^, we crossed *Ripk1*^*fl/fl*^ mice with tamoxifen-inducible *Cd4-Cre*^*ERT2*^ mice (*Ripk1*^*indΔCD4*^ mice). The numbers of different thymocyte populations in *Ripk1*^*indΔCD4*^ mice were compatible with *Ripk1*^*fl/fl*^ littermates after tamoxifen treatment (Fig. 1D), confirming the normal thymopoiesis in the absence of RIPK1 [26] (Fig. 1B). However, similar to *Ripk1*^*ΔCD4*^ mice, we found a significant loss of peripheral naive, effector, and memory CD4^+^ T cells in the spleen of adult *Ripk1*^*indΔCD4*^ mice after 10 weeks of tamoxifen treatment (Fig. 1E), revealing the requirement of RIPK1 for the homeostasis of post-thymic CD4^+^ T cells. The peripheral CD8^+^ T cell numbers were unaffected (Fig. 1E), which could be due to its restriction to the *Cd4*-promotor and the incomplete penetrance of tamoxifen-inducible Cre-lines in T cells [27]. Analysis of naive CD4^+^ and CD8^+^ T cell numbers in the *Ripk1* kinase-dead knock-in mice (*Ripk1*^K45A^) revealed that the scaffold function of RIPK1 is sufficient for peripheral T cell homeostasis and that RIPK1 kinase activity is not implicated (Figs. 1F and S1E).

*Ripk1*^*ΔCD4*^ mice also displayed decreased numbers of peripheral FoxP3^+^ regulatory T cells (Tregs) in the spleen and mLN of *Ripk1*^*ΔCD4*^ mice (Fig. 1G), while *Ripk1*^K45A^ mice had comparable Treg numbers to wild-type littermates (Fig. 1H). Also here, RIPK1 is dispensable for thymic Treg development (Fig. S1F). Furthermore, Treg-specific *Ripk1*-deficient mice (*Ripk1*^*ΔFoxP3*^) also displayed reduced FoxP3^+^ Tregs in the spleen and mLN but expanded numbers of effector T cells (Figs. 1I and S1G, H). These results are in line with a recent report showing that the *Ripk1*^ΔFoxP3^ mice display reduced Treg percentages, eventually leading to fatal systemic autoimmunity [28]. Altogether, our results in *Ripk1*^*ΔCD4*^ mice show the essential role of RIPK1 scaffold function in the homeostasis of naive CD4^+^ and CD8^+^ T cells and FoxP3^+^ Treg cells and in preventing T cell lymphopenia.

### Mixed bone marrow chimeras reveal a survival disadvantage for peripheral RIPK1-deficient T cells

**Fig. 2:**
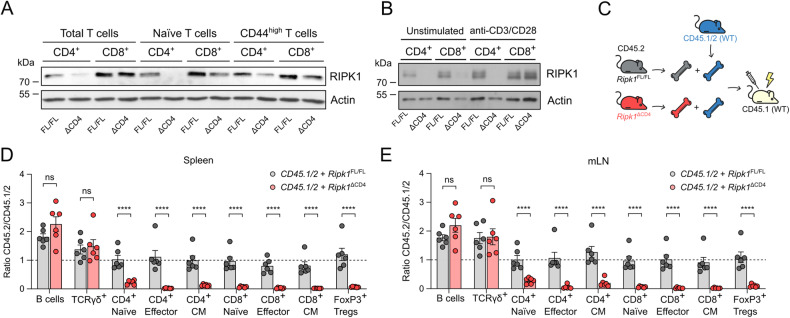
Mixed bone marrow chimeras reveal a survival disadvantage for peripheral RIPK1-deficient T cells. **A**, **B** Western blot analysis of RIPK1 protein levels was performed on CD4^+^ and CD8^+^ T cells, purified by cell sorting from the spleen and lymph nodes of *Ripk1*^ΔCD4^ mice and *Ripk1*^FL/FL^ littermates. **A** Total, naive and CD44^high^, **B** naive T cells, before or after 72 h of stimulation with anti-CD3ε and anti-CD28 antibodies in the presence of IL-2. The following gating strategies were applied: total T cells (live, CD4^+^ or CD8^+^), naive T cells (live, CD4^+^ or CD8^+^, CD25^-^, CD62L^+^ CD44^low^) and CD44^high^ T cells (live, CD4^+^ or CD8^+^, CD62L^−^ CD44^high^). **C** Graphical representation of the mixed bone marrow (BM) chimera setup. CD45.1 recipient mice were sub-lethally irradiated and transfused with a 1:1 mixture of bone marrow cells derived from CD45.1/2 heterozygous wild-type mice and either *Ripk1*^ΔCD4^ or littermate *Ripk1*^FL/FL^ mice (both CD45.2). Bars depicting ratios of CD45.2 to CD45.1/2 from mixed BM chimeras, showing B cells, TCRγδ^+^ T cells, naive, effector, and central memory (CM) CD4^+^ and CD8^+^ T cells, and FoxP3^+^ Tregs isolated from the (**D**) spleen and (**E**) mLN. A ratio of 1 indicates that the specific cell subset was reconstituted equally by the bone marrow cells of both donors. Data are representative of (**B**) two, or (**A**) three independent experiments. **D**, **E** Flow cytometry data are combined from two independent experiments with *n* = 6 mice per group shown as mean ± sem, with each dot representing an individual mouse. *Statistics*: **D**, **E** Statistical significance was calculated by Fisher’s LSD two-way ANOVA on Log_2_-transformed data. ns = non-significant, ****p < 0.0001.

In *Ripk1*^*ΔCD4*^ mice, the floxed *Ripk1* gene is excised upon *Cd4* promotor-controlled Cre recombinase expression. Western blot analysis revealed that peripheral naive CD4^+^ T cells in *Ripk1*^*ΔCD4*^ mice were RIPK1-deficient. In contrast, CD4^+^ effector and central memory (CD44^high^) T cells exhibited residual RIPK1 expression (Fig. 2A). Moreover, some RIPK1 protein was also detected in naive CD8^+^ T cells from *Ripk1*^*ΔCD4*^ mice, while the RIPK1 protein levels in CD44^high^ T cells were only slightly lower than *Ripk1*^*fl/fl*^ littermates (Fig. 2A). We then sorted naive CD4^+^ and CD8^+^ T cells and stimulated them in vitro with anti-CD3 and anti-CD28 antibodies. The CD4^+^ T cells remained RIPK1 deficient before and after stimulation, while naive CD8^+^ T cells exhibited elevated RIPK1 protein levels following anti-CD3/CD28 stimulation. This suggests that some CD8^+^ T cells escapes LoxP recombination and undergo a selective pressure to retain RIPK1 expression following TCR stimulation (Fig. 2B). To further test the survival of *Ripk1*^*ΔCD4*^-derived cells in a competitive environment with wild-type T lymphocytes, we generated mixed bone marrow chimeras. Irradiated CD45.1 recipient mice received bone marrow cells from heterozygous CD45.1/2 allele donors combined with an equal amount of either *Ripk1*^*fl/fl*^ or *Ripk1*^ΔCD4^ bone marrow cells (Fig. 2C). Twelve weeks post bone marrow reconstitution, we found that *Ripk1*^*ΔCD4*^-derived bone marrow cells largely failed to reconstitute CD4^+^ and CD8^+^ naive, effector and central memory T cells, as well as FoxP3^+^ Tregs, in the spleen, mLN and blood (Figs. 2D, E, and S2). However, the *Ripk1*^*ΔCD4*^ bone marrow cells could still reconstitute B cells and unconventional TCRγδ^+^ T cells (Figs. 2D, E, and S2A). These findings demonstrate that *Cd4* promotor-driven *Ripk1*-deficiency result in a competitive survival disadvantage of conventional CD4^+^ and CD8^+^ T cells and FoxP3^+^ Tregs compared to wild-type T cells.

### RIPK1 is essential for survival of naive T cells in a caspase-8-dependent manner

**Fig. 3:**
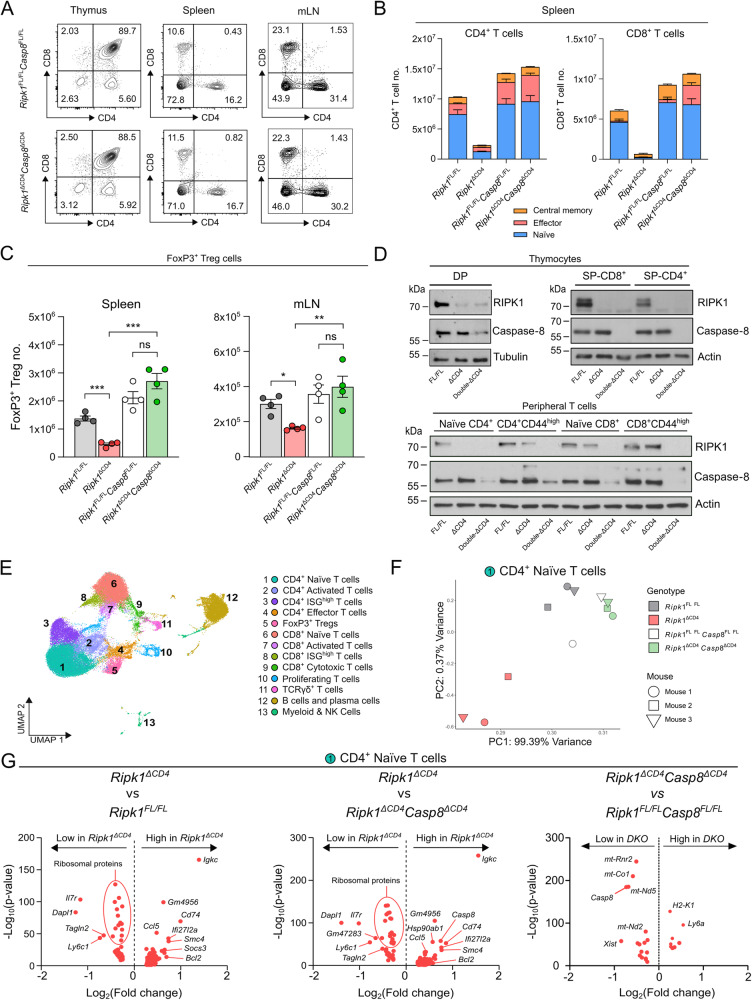
RIPK1 is essential for survival of naive T cells in a caspase-8-dependent manner. **A** Flow cytometry plots of CD4 and CD8 expression in the thymus, spleen, and mLN of *Ripk1*^ΔCD4^*Casp8* ^ΔCD4^ mice and *Ripk1*^FL/FL^*Casp8* ^FL/FL^ littermates representative of at least three independent experiments. Percentage are displayed of each quadrant. **B** Stacked bar plots showing naive, effector and central memory T cells within CD4^+^ (left) and CD8^+^ (right) populations in the spleen of (*n* = 3) *Ripk1*^ΔCD4^ (KO) and (*n* = 4) *Ripk1*^ΔCD4^*Casp8* ^ΔCD4^ (DKO) mice and (*n* = 4) for each of their respective *Ripk1*^FL/FL^ and *Ripk1*^FL/FL^*Casp8* ^FL/FL^ littermates. Data are shown as mean ± sem, and statistical differences are indicated in Table S1. **C** FoxP3^+^ regulatory T cells (Tregs) in the spleen (left) and mLN (right) of *Ripk1*^ΔCD4^ and *Ripk1*^ΔCD4^*Casp8* ^ΔCD4^ mice and their respective *Ripk1*^FL/FL^ and *Ripk1*^FL/FL^*Casp8* ^FL/FL^ littermates. Data are representative of three independent experiments and shown for *n* = 4 mice per group. (**D**, upper panels) Western blot analysis of RIPK1 and caspase-8 protein levels was performed on (Upper) CD4^+^CD8^+^ double positive (DP), CD4^+^ single positive and CD8^+^ single positive thymocytes, purified by cell sorting from the thymus of *Ripk1*^ΔCD4^ mice, *Ripk1*^FL/FL^ littermates and *Ripk1*^ΔCD4^*Casp8* ^ΔCD4^ mice. The following gating strategies were applied: DP (live, CD3^-^CD4^+^CD8^+^TCR^-^), CD4^+^ (live, Lin^−^ CD3^+^ CD4^+^CD8^−^ TCRβ^+^), and CD8^+^ (live, Lin^-^ CD3^+^ CD4^−^CD8^+^ TCRβ^+^). (**D**, lower panel) Western blot analysis performed on peripheral CD4^+^ and CD8^+^ T cells (naive and CD44^high^), purified cell sorting from the combined spleens and lymph nodes of *Ripk1*^ΔCD4^ mice, *Ripk1*^FL/FL^ littermates and *Ripk1*^ΔCD4^*Casp8* ^ΔCD4^ mice. The following gating strategies were applied: naive T cells (live, CD4^+^ or CD8^+^, CD25^-^, CD62L^+^ CD44^low^) and CD44^high^ T cells (live, CD4^+^ or CD8^+^, CD62L^−^ CD44^high^). **E** Cells were isolated from the mLN of *Ripk1*^ΔCD4^ (*n* = 3) mice, *Ripk1*^ΔCD4^*Casp8* ^ΔCD4^ (*n* = 3) mice and their respective *Ripk1*^FL/FL^ (*n* = 3) and *Ripk1*^FL/FL^*Casp8* ^FL/FL^ (*n* = 2) littermates and subjected to single-cell RNA sequencing (scRNA-seq). Clusters of CD4^+^ and CD8^+^ T cells, TCRγδ^+^ T cells, B cells, proliferating cells, and myeloid cells were identified (Fig. S3F) and presented in a Uniform Manifold Approximation and Projection (UMAP) plot. **F** Principal component analysis (PCA) showing the average gene expression in CD4^+^ naive T cells (Cluster 1) for the individual *Ripk1*^ΔCD4^ and *Ripk1*^ΔCD4^*Casp8* ^ΔCD4^ mice and their respective *Ripk1*^FL/FL^ and *Ripk1*^FL/FL^*Casp8* ^FL/FL^ littermates. **G** Volcano plots representing the differentially expressed (DE)-genes in the cluster of CD4^+^ naive T cells (C1) of *Ripk1*^ΔCD4^ mice compared to *Ripk1*^FL/FL^ littermates (left), *Ripk1*^ΔCD4^ mice compared to *Ripk1*^ΔCD4^*Casp8* ^ΔCD4^ mice (middle) or *Ripk1*^ΔCD4^*Casp8* ^ΔCD4^ mice compared to *Ripk1*^FL/FL^*Casp8* ^FL/FL^ littermates (right). Significance (-Log_10_ of the *p*-value) is indicated on the y-axis and Log_2_ of the fold-change in gene expression is indicated on the x-axis. Data are representative of (**D**) two, (**A**) three, or (**B**) four independent experiments. *Statistics*: statistical significance was calculated by (**D**) Fisher’s LSD one-way ANOVA. ns = non-significant, *p < 0.05, **p < 0.01, ***p < 0.001.

To examine whether the induction of extrinsic apoptosis affects the survival of RIPK1 deficient T cells, we created mice with a T cell-specific deletion of both *Ripk1* and *Casp8* (*Ripk1*^*ΔCD4*^*Casp8* ^*ΔCD4*^). Similar as in *Ripk1*^*ΔCD4*^ mice (Fig. 1A, B), no significant differences were found in percentages and numbers of the different thymic subsets between *Ripk1*^*ΔCD4*^*Casp8* ^*ΔCD4*^ mice and *Ripk1*^*fl/fl*^*Casp8* ^*fl/fl*^ littermates (Figs. 3A, and S3A, B), excluding a role for caspase-8. Genetic removal of *Casp8* in *Ripk1*-deficient T cells resulted in comparable percentages and total numbers of both CD4^+^ and CD8^+^ peripheral T cells in spleen and mLN similar to littermate controls (Figs. 3A, B, S3C, D, and Table S1). Suggesting that RIPK1 deficient T cells die by caspase-8-mediated apoptosis in the periphery. Additionally, while CD8^+^ naive and central memory T cell numbers were restored in *Ripk1*^*ΔCD4*^*Casp8* ^*ΔCD4*^ mice, increased numbers of CD8^+^ effector T cells were observed in both spleen and mLN compared to littermate controls (Figs. 3B and S3D). In addition, the total number of FoxP3^+^ Tregs were likewise rescued in both spleen and mLN of *Ripk1*^*ΔCD4*^*Casp8* ^*ΔCD4*^ mice (Fig. 3C). Western blot analysis revealed that in thymic subsets, Ripk1^*ΔCD4*^ and *Ripk1*^*ΔCD4*^*Casp8* ^*ΔCD4*^ mice exhibit similar efficiency in Cre-mediated *Ripk1* ablation. DP *Ripk1*^*ΔCD4*^ and *Ripk1*^*ΔCD4*^*Casp8* ^*ΔCD4*^ thymocytes showed some residual RIPK1 and caspase-8 protein expression, whereas SP-CD4^+^ or SP-CD8^+^ thymocytes displayed potent RIPK1 and caspase-8 deletion (Fig. 3D). Similarly, RIPK1 and caspase-8 protein levels were undetectable in peripheral CD4^+^ and CD8^+^ T cells from *Ripk1*^*ΔCD4*^*Casp8* ^*ΔCD4*^ mice (Fig. 3D). Indeed, enhanced caspase-8 cleavage could be observed in peripheral naive CD4^+^ *Ripk1*^*ΔCD4*^ T cells (Fig. S3E). Altogether, these data demonstrate that peripheral T cell lymphopenia due to the absence of RIPK1 scaffold function is caused by caspase-8-mediated apoptosis.

### RIPK1-deficient T cells display transcriptome adaptations that are reverted by additional Casp8 gene deletion

To explore how the loss of RIPK1 affected T cell function and how the absence of caspase-8 regulated the survival of RIPK1 deficient T cells, we performed single-cell RNA sequencing (scRNA-seq) on cells isolated from the mLN and enriched for T cells from *Ripk1*^*ΔCD4*^ and *Ripk1*^*ΔCD4*^*Casp8* ^*ΔCD4*^ mice along with their respective littermate controls. Based on cell-specific annotation markers (Fig. S3F), we identified different CD4^+^ and CD8^+^ T cell subsets among the different immune subsets (Figs. 3E and S3F). As naive CD4^+^ T cells were consistently RIPK1 deficient in both *Ripk1*^*ΔCD4*^ and *Ripk1*^*ΔCD4*^*Casp8* ^*ΔCD4*^ mice, we focused the scRNA-seq analysis on this cluster (Fig. 3E). Principle component analysis (PCA) of average gene expression in naive CD4^+^ T cells revealed a significant variation between *Ripk1*^*ΔCD4*^ mice and *Ripk1*^*fl/fl*^ littermates (Fig. 3F). Interestingly, in naive CD4^+^ T cells no variation was observed between *Ripk1*^*ΔCD4*^*Casp8* ^*ΔCD4*^ mice and littermate controls (Fig. 3F), indicating an overall transcriptional rescue of the *Ripk1*^*ΔCD4*^ phenotype by *Casp8-*deficiency. Volcano plots of the differentially expressed (DE) genes revealed that naive CD4^+^ T cells from *Ripk1*^*ΔCD4*^ mice and *Ripk1*^*ΔCD4*^*Casp8* ^*ΔCD4*^ mice had almost identical DE genes as *Ripk1*^*ΔCD4*^ mice and *Ripk1*^*fl/fl*^ littermates (Fig. 3G, left and middle panels). Indeed, surprisingly few transcriptional differences were observed between double-deficient *Ripk1*^*ΔCD4*^*Casp8* ^*ΔCD4*^ and wild-type littermates (Fig. 3G, right panel). Interestingly, *Il7r* and *Bcl2* were two of the most prominent DE genes, with *Il7r* expression being decreased and *Bcl2* expression being increased in *Ripk1*^*ΔCD4*^ mice. The B cell/plasma cell markers *Igkc* and *Cd74* were upregulated in all examined immune cell populations of *Ripk1*^*ΔCD4*^ mice (Figs. 3G, and S4A, B), showing that their upregulation is not limited to T cells. PCA plots of the B cell/plasma cell cluster further revealed no transcriptomic differences or alterations in the pan-B cell marker *Cd19* between genotypes (Figs. S4C, D). Conversely, similar to RIPK1-deficient naive CD4^+^ T cells, PCAs revealed transcriptomic alterations in all identified CD4^+^ and CD8^+^ T cell clusters between *Ripk1*^*ΔCD4*^ mice and wild-type littermates, which were reverted by additional caspase-8 deletion (Fig. S4D). These results show a complete cellular and transcriptional rescue of the *Ripk1*^*ΔCD4*^ T cell phenotypes by additional caspase-8 deletion.

### RIPK1-deficient naive T cells proliferate but die following activation due to extrinsic caspase-8-dependent apoptosis

**Fig. 4:**
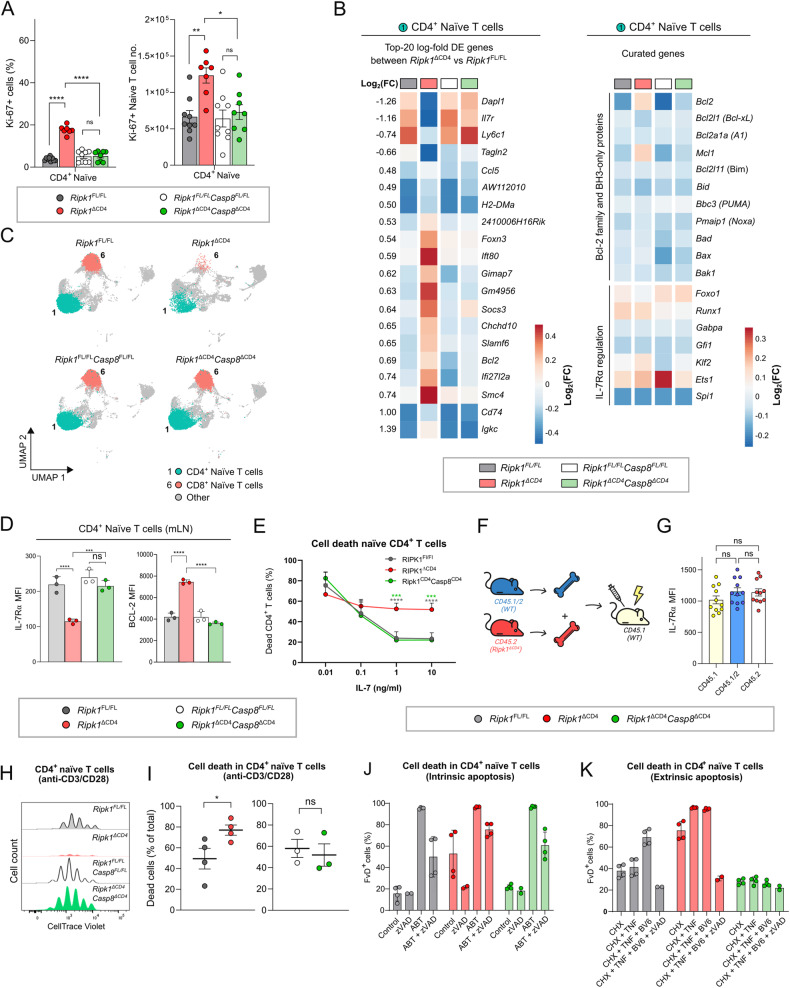
RIPK1-deficient naive T cells proliferate but die following activation due to extrinsic caspase-8-dependent apoptosis. **A** Bar plots showing percentages (left) and absolute numbers (right) of Ki-67^+^ cells within the population of CD4^+^ naive T cells in the mLN of (*n* = 7) *Ripk1*^ΔCD4^ mice and (*n* = 8) *Ripk1*^ΔCD4^*Casp8* ^ΔCD4^ mice, compared with (*n* = 9) *Ripk1*^FL/FL^ and (*n* = 8) *Ripk1*^FL/FL^*Casp8* ^FL/FL^ littermates. Results are combined from two independent experiments. **B** Heatmaps displaying the relative average expression levels of the top-20 DE genes (left) and curated genes (right) in CD4^+^ naive T cells (Cluster 1, Fig. 4C) of *Ripk1*^ΔCD4^*Casp8* ^ΔCD4^ (*n* = 3) mice and their respective *Ripk1*^FL/FL^ (*n* = 3) and *Ripk1*^FL/FL^*Casp8* ^FL/FL^ (*n* = 2) littermates. Average gene expression shown as Log_2_ Fold-change (FC) is indicated for each genotype in columns, and rows represent individual genes. **C** UMAP plot indicating the clusters of CD4^+^ naive (Cluster 1, cyan) and CD8^+^ naive (Cluster 6, red) T cells separated over the four different genotypes. **D** Bar graphs indicating protein levels of surface IL-7Rα (left) and intracellular BCL-2 (right), represented as the mean fluorescent intensity (MFI), in CD4^+^ naive T cells isolated from the mLN. Data are representative of (D-left) three independent experiments with *n* = 3 mice per group, (D-right) one experiment with *n* = 3 mice per group. **E** CD4^+^ naive T cells were purified from the spleen and lymph nodes by cell sorting and stimulated during 72 h with different concentrations of IL-7. Cell death was determined by flow cytometry and represented as the percentage of dead cells within the CD4^+^ T cell population. Data are combined from *n* = 6 (*Ripk1*^FL/FL^), or *n* = 3 (*Ripk1*^ΔCD4^ and *Ripk1*^ΔCD4^*Casp8* ^ΔCD4^) biological replicates. **F** Graphical representation of the mixed bone marrow (BM) chimera setup. **G** Bar graphs indicating surface protein levels of IL-7Rα, represented as the mean fluorescent intensity (MFI), in CD4^+^ naive T cells from the spleen of mixed bone marrow chimera mice. Data are pooled from three independent experiments with *n* = 11 mice. **H**, **I** CD4^+^ naive T cells were purified from the spleen and mLN by cell sorting, labeled with CellTrace Violet, and stimulated for 72 h with anti-CD3ε and anti-CD28 antibodies in the presence of IL-2. **H** Representative proliferation plots of CD4^+^ T cells is shown by the reduction in CellTrace Violet staining for each division. **I** Percentages of dead CD4^+^ T cells were determined in every independent experiment by flow cytometry after 72 h. Data are combined from (I-right) *n* = 3 or (I-left) *n* = 4 independent biological experiments. **J**, **K** CD4^+^ naive T cells were purified from the spleen and lymph nodes by cell sorting, pre-treated with different combinations of cycloheximide (CHX), BV6, or zVAD for 30 min at 37 °C and 5% CO_2_, and subsequently stimulated with ABT-737 or TNF for 20 h. Cell death was determined by flow cytometry and represented as the percentage of dead cells within the naive CD4^+^ T cell population based on fixable viability dye (FvD) staining. **J**, **K** Data are combined from two independent experiments with *n* = 4 biological replicates or *n* = 2 in the (**J**; zVAD) condition and *n* = 2 in the (**K**; CHX + TNF + BV6 + zVAD) condition. **A**, **D**, **G**–**K** Flow cytometry data are shown as (**A**, **D**, **G**, **I**) mean ± sem, or (**E**, **J**, **K**) mean ± SD, and each dot (**E**) represents the mean, or (**A**, **D**, **G**, **I**) or an individual mouse or biological replicate. *Statistics*: Statistical significance was calculated by (**A**, **D**, **G**) Fisher’s LSD one-way ANOVA on absolute values or (**A**) Log_2_-transformed data, (**I**) unpaired *t*-test (two-sided) on absolute values or (**E**, **J**, **K**) Fisher’s LSD two-way ANOVA on absolute values. ns = non-significant, *p < 0.05, **p < 0.01, ***p < 0.001, ****p < 0.0001.

The T cell lymphopenic phenotype observed in the *Ripk1*^*ΔCD4*^ mice could indicate defective homeostatic proliferation. However, despite dramatically reduced absolute numbers of naive peripheral T cells, we observed increased amounts and frequencies of Ki-67^+^ naive CD4^+^ T cells in the mLN of *Ripk1*^*ΔCD4*^ mice compared to *Ripk1*^*fl/fl*^ littermates (Figs. 4A and S4E). The increased Ki-67^+^ expression suggests the occurrence of enhanced proliferation as a compensatory mechanism to replenish the T cell numbers following severe T cell lymphopenia [29]. This phenomenon does not happen in *Ripk1*^*ΔCD4*^*Casp8* ^*ΔCD4*^ mice, revealing a restoration of the proliferation phenotype by the absence of the *Casp8* gene (Figs. 4A and S4E). We depicted the top-20 DE genes based on the Log_2_ fold-change in expression (Figs. 4B, C, S4F). As naive T cells depend on IL-7 signaling for survival [30], and BCL-2 limits mitochondrial apoptosis, we further investigated anti-apoptotic *Bcl2* family members and IL-7Rα regulators. Of the *Bcl2* family members, only the anti-apoptotic *Bcl2* and *Mcl1* were enhanced in *Ripk1*^*ΔCD4*^ mice, while the pro-apoptotic Bcl2 family members remained unchanged (Fig. 4B). Similarly, the expression of key IL-7Rα regulators (*Foxo1*, *Runx1*, *Gabpa*, *Gfi1*, *Klf2*, *Ets1* and *Spi1*) remained unchanged in naive CD4^+^ T cells of *Ripk1*^*ΔCD4*^ mice (Fig. 4B), suggesting that the *Il7r* downregulation is not caused by altered expression of these transcription factors. Additionally, we validated the reduced IL-7Rα surface expression and increased BCL-2 protein levels in naive CD4^+^ T cells by flow cytometry in *Ripk1*^*ΔCD4*^ mice (Fig. 4D). Measuring the effect of IL-7 stimulation on RIPK1 deficient T cell survival shows that even at higher IL-7 concentrations RIPK1 deficiency resulted in reduced survival responses, which was entirely rescued in the *Ripk1*^*ΔCD4*^*Casp8* ^*ΔCD4*^ mice (Figs. 4E and S4G). Furthermore, despite reduced numbers of *Ripk1*^*ΔCD4*^ naive CD4^+^ T cells in bone marrow chimeras (Fig. 2D, E), their IL-7Rα expression was restored in the presence of wild-type cells, indicating this reduction is be induced by the environment (Fig. 4F, G).

Anti-CD3/CD28 stimulation of naive CD4^+^ and CD8^+^ T cells showed that *Ripk1*^*ΔCD4*^ cells were capable of proliferating but displayed enhanced cell death following the TCR stimulation (Figs. 4H, I, S4H, S4I). Previously, T cells from *Casp8* ^*ΔCD4*^ mice were reported to display enhanced RIPK1-dependent necroptosis in response to anti-CD3/CD28 stimulation [31]. However, both the enhanced cell death and proliferative capabilities were the same between cells from *Ripk1*^*ΔCD4*^*Casp8* ^*ΔCD4*^ mice and their wild-type littermates (Fig. 4H, I), showing a complete rescue of the anti-CD3/CD28 induced cell death in *Ripk1*^*ΔCD4*^ T cells by additional *Casp8* deletion. Similarly, the spontaneous cell death of cultured naive CD4^+^ T cells from *Ripk1*^*ΔCD4*^ mice could be rescued by the addition of caspase-inhibitor zVAD-fmk alone (Fig. 4J). Whereas, naive CD4^+^ T cells from *Ripk1*^*ΔCD4*^*Casp8* ^*ΔCD4*^ mice were not protected from ABT-737-triggered mitochondrial apoptosis (Fig. 4J), confirming intrinsic apoptosis to be caspase-8 independent in T cells. Accordingly, we found that naive CD4^+^ T cells from *Ripk1*^*ΔCD4*^ mice were more sensitive to TNF-mediated cell death both in the presence and absence of the Smac mimetic BV6 (Fig. 4K), showing that *Ripk1* gene ablation leads to enhanced TNF-induced extrinsic apoptosis [32, 33]. The addition of zVAD-fmk rescued this, and no enhanced cell death was observed in any condition with *Ripk1*^*ΔCD4*^*Casp8* ^*ΔCD4*^ cells (Fig. 4K). Combined, these data confirm that the enhanced cell death in naive CD4^+^ T cells from *Ripk1*^*ΔCD4*^ mice is mediated by caspase-8-dependent apoptosis, possibly through TNF engagement.

### Naive T cells and FoxP3^+^ Tregs require RIPK1 for protection from TNFR1-induced apoptosis

**Fig. 5:**
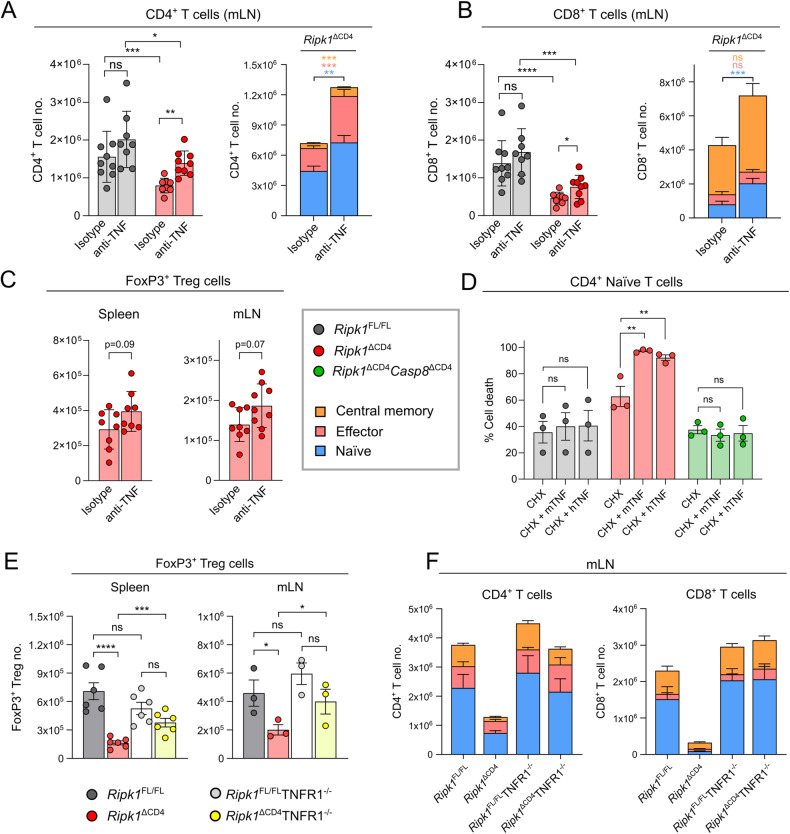
Naive T cells and FoxP3^+^ Tregs require RIPK1 for protection from TNFR1-induced apoptosis. **A**, **B** *Ripk1*^ΔCD4^ mice and *Ripk1*^FL/FL^ littermates were treated with anti-TNF-α antibodies or isotype control every 3–4 days for two weeks. Total numbers of (A-left) CD4^+^ or (B-left) CD8^+^ T cells and stacked bar plots divided into naive (CD62L^+^CD44^low^), effector (CD62L^−^CD44^high^), and central memory (CD62L^+^CD44^high^) (**A**-right) CD4^+^ and (**B**-right) CD8^+^ T cells in the mLN. Data are combined from two independent experiments with *n* = 8 in the *Ripk1*^*FL/FL*^ (Isotype) and *Ripk1*^*ΔCD4*^ (α-TNF) groups and *n* = 9 mice in the *Ripk1*^*ΔCD4*^ (Isotype) and *Ripk1*^*FL/FL*^ (α-TNF) groups. **C** FoxP3^+^ regulatory T cells (Tregs) in the spleen (left) and mLN (right) of *Ripk1*^ΔCD4^ mice and *Ripk1*^FL/FL^ littermates treated with anti-TNF-α antibodies or isotype control. Data are combined from two independent experiments with *n* = 8 mice per group. **D** CD4^+^ naive T cells were purified by cell sorting from the spleen and lymph nodes of *Ripk1*^ΔCD4^ mice, *Ripk1*^FL/FL^ littermates, and *Ripk1*^ΔCD4^*Casp8* ^ΔCD4^ mice, and pre-treated with cycloheximide and stimulated during 20 h with mTNF or hTNF. Cell death was determined by flow cytometry and represented as the percentage of dead cells within the CD4^+^ T cell population. Data are combined and shown for *n* = 3 independent biological experiments. **E** FoxP3^+^ regulatory T cells (Tregs) in the spleen (*n* = 6) and mLN (*n* = 3) of *Ripk1*^ΔCD4^ and *Ripk1*^ΔCD4^TNFR1^−/−^ mice and their respective *Ripk1*^FL/FL^ and *Ripk1*^FL/FL^TNFR1^−/−^ littermates. Data are representative of two independent experiments. **F** Stacked bar plots showing naive, effector, and central memory T cells within CD4^+^ (left) and CD8^+^ (right) populations in the mLN of *Ripk1*^ΔCD4^ and *Ripk1*^ΔCD4^TNFR1^−/−^ mice and their respective *Ripk1*^FL/FL^ and *Ripk1*^FL/FL^TNFR1^−/−^ littermates. Data are representative of two independent experiments and shown for *n* = 3 mice per group. Statistical data for each population is shown in Table S2. **A**–**F** Flow cytometry data are shown as mean ± sem, and **A**–**E** each dot representing an individual mouse or (**D**) the average of technical replicates in an independent experiment. *Statistics*: Statistical significance was calculated by (**C**) unpaired *t*-tests (two-sided), or (**D**) Fisher’s LSD two-way ANOVA on absolute values, or (**E**) Fisher’s LSD one-way ANOVA, or (**A**, **B**, **F**) Log_2_-transformed data. ns = non-significant, *p < 0.05, **p < 0.01, ***p < 0.001, ****p < 0.0001.

We blocked endogenous TNF in *Ripk1*^*ΔCD4*^ mice to follow up on this finding and since in vivo anti-TNF blockade was reported to restore hematopoietic reconstituted RIPK1-deficient T cells in the bone marrow [23]. TNF blockade by administration of anti-TNF antibodies every 3–4 days during two weeks increased the absolute numbers of naive, effector and central memory CD4^+^ T cells and naive CD8^+^ T cells in both spleen and mLN, while no difference was observed in *Ripk1*^*fl/fl*^ littermates (Figs. 5A, B and S5A, B). Furthermore, Treg numbers were not significantly increased, showing them to be less affected by short time anti-TNF treatment than conventional CD4^+^ and CD8^+^ T cell numbers (Fig. 5C).

Murine TNF (mTNF) engages both TNFR1 and TNFR2, while human TNF (hTNF) is a specific agonist for murine TNFR1 [34]. While the numbers of SP thymocytes were unchanged in *Ripk1*^*ΔCD4*^ mice (Fig. 1B), exposure to mTNF and hTNF of both thymic (Fig. S5C) and peripheral naive CD4 T cells (Fig. 5D) resulted in increased cell death. Additional deletion of caspase-8 rescued this phenotype (Figs. 5D, S5C). This indicates that *Ripk1*-deficiency sensitizes both CD4^+^ thymocytes and peripheral naive CD4+ T cells to TNFR1-induced, caspase-8-dependent extrinsic apoptosis.

Therefore, we generated mice with an additional deletion of TNFR1 (*Ripk1*^*ΔCD4*^TNFR1^−/−^) to determine the in vivo contribution of TNFR1-mediated apoptosis to the T cell lymphopenia of *Ripk1*^*ΔCD4*^ mice. In line with our previous data from *Ripk1*^*ΔCD4*^*Casp8* ^*ΔCD4*^ mice, the reduced numbers of FoxP3^+^ Tregs naive, effector, and central memory CD4^+^ and CD8^+^ T cells in *Ripk1*^*ΔCD4*^ mice were rescued by additional deletion of TNFR1 in both spleen and mLN (Figs. 5E, F S5D, and Table S2). These results demonstrate that the peripheral T cell lymphopenia in *Ripk1*^*ΔCD4*^ mice can be attributed to an increased sensitivity of RIPK1-deficient T cells to caspase-8 and TNFR1-mediated apoptosis.

## Discussion

Peripheral T cell homeostasis demands an exact balance between T cell survival and cell death at the different stages of T cell maturation to ensure an appropriate immune response and remove the effector T cells after clonal expansion [35, 36]. RIPK1 deficiency was previously reported to result in the loss of peripheral T cells [24, 25], but there is limited knowledge on the requirement of RIPK1 in different peripheral T cell populations. We found that *Ripk1*^ΔCD4^ mice display severe peripheral T cell lymphopenia, which is primarily mediated by a loss of naive CD4^+^ and CD8^+^ T cells, though cell numbers in the thymus were not reduced. The latter confirms previous reports that despite thymic expression of RIPK1, normal thymopoiesis occurs in the absence of RIPK1 [26]. Interestingly, unlike *Ripk1*^*Cd4-cre*^ mice, early hematopoiesis in the thymus and bone marrow was abrogated in *Ripk1*^*iVav-cre*^ mice and rescued with additional *Mlkl* deficiency [37]. These data indicate that RIPK1-deficient hematopoietic stem cells are more susceptible to IFNγ and ZBP1-dependent necroptosis in the bone marrow and thymus. In contrast, splenic CD4 and CD8 T cells from *Ripk1*^*iVav-cre*^ mice were not rescued by additional MLKL deficiency [37], which supports our findings that peripheral RIPK1-deficient T cells undergo TNFR1-dependent apoptosis.

In mice the peripheral naive T cell pool primarily maintains its numbers by a continuous replenishment from the thymus, although lymphopenia can enhance peripheral homeostatic proliferation [29, 38, 39]. Western blots revealed that low amounts of RIPK1 remain expressed in the DP thymocytes from *Ripk1*^ΔCD4^ mice, which is consistent with previous reports on *Cd4*-*Cre* line’s activation profile at the DP stage [40]. Nevertheless, SP-CD4 and SP-CD8 thymocytes and peripheral naive CD4 T cells displayed effective RIPK1 deletion. Contrary to thymic SP-CD8 cells, peripheral naive CD8^+^ T cells expressed low amounts of RIPK1. However, following in vitro anti-CD3/CD28 stimulation, the CD8^+^ T cells displayed RIPK1 levels comparable with those in wild-type, indicating a selective pressure to retain RIPK1 expression to survive following activation. Despite the incomplete deletion of RIPK1 in the remaining peripheral CD8^+^ T cells, the CD8^+^ T cell compartment was widely reduced in *Ripk1*^*ΔCD4*^ mice. Similarly, *Ripk1*^ΔCD4^-derived bone marrow cells failed to reconstitute any population of conventional naive, effector, and central memory CD4^+^ and CD8^+^ T cells or Tregs in the presence of wild-type cells, demonstrating a competitive survival disadvantage of these RIPK1-deficient T cell subsets.

Kinase-dead *Ripk1*^K45A^ mice did not display any change in total T cell numbers, confirming earlier findings [24]. Further subset analysis clarified that the RIPK1 scaffold function, rather than the kinase activity, is crucial for the steady state homeostasis of peripheral naive, effector, or central memory CD4^+^ and CD8^+^ T cells. In addition, we found that peripheral FoxP3^+^ Tregs also depend on the RIPK1-scaffold function to avoid caspase-8 mediated apoptosis, while RIPK1 deficiency did not affect the numbers of thymic FoxP3^+^ Tregs.

In order to identify the mechanism of cell death implicated in the T cell lymphopenia, we showed that additional caspase-8 deletion in *Ripk1*^*ΔCD4*^*Casp8* ^*ΔCD4*^ mice resulted in a complete restoration of the T cell numbers with similar single-cell transcriptomic profiles as wild-type littermates. Upon in vitro anti-CD3/CD28 stimulation, naive CD4^+^ T cells from *Ripk1*^*ΔCD4*^*Casp8* ^*ΔCD4*^ mice proliferate and die to the same extent as wild-type littermates. Because the absence of caspase-8 sensitizes T cells to RIPK1-dependent necroptosis [8, 10, 31], the additional loss of RIPK1 seems to completely block the switch to necroptosis in T cells of *Ripk1*^*ΔCD4*^*Casp8* ^*ΔCD4*^ mice. This indicates that the absence of both RIPK1 and caspase-8 rescues each other’s T cell phenotypes and implies that at steady state, neither T cell development nor peripheral T cell homeostasis requires RIPK1 or caspase-8 as long as both are deleted. At steady-state, percentages and numbers of *Ripk1/Casp8* double-deficient T cell populations are similar as wild-type T cells. However, whether the absence of both genes still allows for an appropriate T-cell response during infection in vivo remains to be explored.

We found that the increased apoptosis of peripheral T cells in *Ripk1*^ΔCD4^ mice was associated with enhanced markers of proliferation in the naive T cells, likely constituting a form of lymphopenia-induced proliferation [29, 38]. This compensatory proliferation was associated with decreased expression of IL-7Rα and IL-7 responsiveness in naive CD4^+^ cells from *Ripk1*^ΔCD4^ mice, which was reverted in *Ripk1*^*ΔCD4*^*Casp8* ^*ΔCD4*^ mice. Two recent studies showed that T cell-specific RIPK1-deficient mice display characteristics of senescent cells and that this is associated with the development of age-related inflammatory diseases in mostly 7–12 months-old mice [25, 41]. While one study reported that the age-related pathologies were FADD-dependent [41], the other study showed that it was associated with increased mTOR activity [25]. However, transferring RIPK1-deficient T cells into a wild-type environment partially reduced the hyperactive mTOR phenotype, suggesting that the enhanced mTOR activity is a cell-extrinsic effect of RIPK1-deficiency [25]. In light of the cellular rescue by additional caspase-8 deletion, the reported senescent T cell phenotype following RIPK1 deficiency [25] may be caused by the lymphopenia-induced dysbalance leading to age-related sequelae.

By use of several mouse models, we show that the RIPK1 scaffold function protects naive, effector and central memory CD4^+^ and CD8^+^ T cells, as well as FoxP3^+^ regulatory T cells, from caspase-8-mediated apoptosis in vivo. Moreover, *Ripk1*-deficient naive CD4^+^ T cells display an increased sensitivity to TNFR1-induced apoptosis following TNF stimulation in vitro. TNF has previously been implicated in the death of RIPK1-deficient T cells in the bone marrow during hematopoietic reconstitution experiments [23]. Accordingly, specific deletion of TNFR1 in *Ripk1*^*ΔCD4*^*Tnfr1*^*−/−*^ mice revealed that naive T cells and FoxP3^+^ Tregs require RIPK1 to avoid TNFR1-induced apoptosis in vivo. These findings provide novel insights into T cell survival mechanisms and emphasize the essential role of the RIPK1 in peripheral T cell homeostasis of not only FoxP3^+^ Tregs but also conventional naive, effector, and memory T cells and, could provide important insights for therapeutic targeting of RIPK1 in T cell-driven diseases.

## Materials and methods

### Mice

Conditional *Ripk1* mice (*Ripk1*^FL/FL^) were generated in-house as described before [27]. *Cd4-Cre* transgenic mice were generated as described before [35]. T-cell-specific *Ripk1*-knockout mice (*Ripk1*^ΔCD4^) were created by crossing *Ripk1*^FL/FL^ mice with *Cd4-Cre* transgenic mice. Mice were bred by crossing heterozygous parents (*Ripk1*^FL/+^ *Cd4-Cre*^Tg/+^) with *Ripk1*^FL/FL^ mice. *Ripk1*^FL/+^ *Cd4-Cre*^+/+^ and *Ripk1*^FL/FL^ *Cd4-Cre*^+/+^ mice were used as littermate controls. Tamoxifen-inducible T cell specific *Ripk1*-knockout mice (*Ripk1*^indΔCD4^) were created by crossing *Ripk1*^FL/FL^ mice with *Cd4-Cre*-ER^T2^ transgenic mice (Jackson Laboratory, Bar Harbor, ME, USA). For in vivo *Ripk1* deletion, *Ripk1*^indΔCD4^ mice were injected intraperitoneally three consecutive days with 1 mg tamoxifen (T5648, Sigma-Aldrich, Burlington, MA, USA) dissolved in corn oil (C8267, Sigma-Aldrich) followed by a single injection every 2 weeks during 10 weeks before being used for experiments. Alternatively, the mice were fed with a tamoxifen-containing diet (TD.55125.I 400 mg/kg, Envigo, Indianapolis, IN, USA) during 10 weeks. RIPK1 kinase-dead knock-in mice (*Ripk1*^K45A^) were described before [36] and purchased from GlaxoSmithKline (GSK, London, UK). *FoxP3-Cre* transgenic mice were kindly provided by Prof. Dr. Bart Lambrecht (Ghent University, Belgium). Treg-specific *Ripk1*-knockout mice (*Ripk1*^ΔFoxP3^) were created by crossing *Ripk1*^FL/FL^ with *FoxP3-Cre* transgenic mice. CD45.1 and CD45.1/2 mice were from the Jackson Laboratory. Conditional *Casp8* mice (*Casp8* ^FL/FL^) were described before [37]. T cell-specific *Ripk1-* and *Casp8-*double knockout mice (*Ripk1*^ΔCD4^*Casp8* ^ΔCD4^) were created by crossing *Casp8* ^FL/FL^ mice with *Ripk1*^FL/+^ *Cd4-Cre*^Tg/+^ mice. TNFR1-deficient mice (*Tnfr1*^−/−^) were described before [38]. Full-body *Tnfr1*-deficient and T cell-specific *Ripk1*-deficient mice were created by crossing *Tnfr1*^−/−^ mice with *Ripk1*^FL/+^ *Cd4-Cre*^Tg/+^ mice. All mice were bred and housed in individually ventilated cages at the VIB Center for Inflammation Research under specific pathogen-free (SPF) conditions in a temperature-controlled (21 °C) animal facility with a 14/10-h light/dark cycle. Water and food were provided *ad libitum*. Mice were used for experiments at an age of 8–12 weeks unless otherwise specified. Researchers were not blinded. Within each genotype, mice were randomly assigned to anti-TNF or isotype control groups. Irradiated mice were randomly assigned to the individual bone marrow chimera groups. Effect size and sample size calculation was performed using G*power 3.1.9.7. software with an α-error of 0.05 and a power of 95%. The number of mice (*n*) for each experiment is reported in the figure legends. Both male and female mice were used for all the experiments. Only male Treg-specific *Ripk1*-knockout mice (*Ripk1*^ΔFoxP3^) were used as the transgene in these mice is located on the X-chromosome and suffers from X-chromosome inactivation in female mice. For in vivo neutralization of TNF-α, mice received doses of anti-TNF-α (MP6-XT22, Biolegend, San Diego, CA, USA) or IgG isotype control (RTK2071, Biolegend) every 3–4 days during 2 weeks by intraperitoneal injection (4 µg/g body weight). Afterwards, cells were collected as described below and T cell numbers were measured by flow cytometry.

All experiments on mice were conducted in accordance with the Declaration of Helsinki and approved by the IRC-UGent ethical committee under the reference numbers (EC2020-045 and EC2022-078).

### Genotyping

Genotyping of the different mouse lines was performed by PCR. Wild type and *loxP*-site flanked *Ripk1* alleles were identified using the following PCR primers: 5′-GGCAAACACCTTTAATCCAAGCCTGGTC-3′, 5′-GGCAAACACCTTTAATCCAAGCCTGGTC-3′ and 5′-CCATGGCTGCAAACACCTAAACCTGAAG-3′, yielding a 287 bp wild-type DNA fragment and a 366 bp floxed DNA fragment. The *Cd4-Cre* construct was identified using 2 additional primers; 5′-GCCTGCATTACCGGTCGATGCAACGA-3′ and 5′-GTGGCAGATGGCGCGGCAACACCAT-3′, resulting in an 800 bp DNA fragment when the transgene is present. *Ripk1*^K45A^ mice were identified using the 5′-CTCTGATTGCTTTATAGGACACAGCA-3′ forward and 5′-GTCTTCAGTGATGTCTTCCTCGTA-3′ reverse primers, yielding a 575 bp wild-type DNA fragment and a 473 bp knock-in DNA fragment. The presence of the transgenic *Foxp3-Cre* construct was detected using a combination of the following PCR primers: 5′-CCTAGCCCCTAGTTCCAACC-3′, 5′-AAGGTTCCAGTGCTGTTGCT-3′, 5′-CCTGGTGATGAGGAGAATCAG-3′ and 5′-ATTTCAGGGATGGACACACC-3′, yielding a 322 bp wild-type DNA fragment and a 585 bp transgenic DNA fragment. Identification of the *loxP*-site flanked C*asp8* alleles was performed by using the following PCR primers: 5′-TTGAGAACAAGACCTGGGGACTG-3′ and 5′-GGATGTCCAGGAAAAGATTTGTGTC-3′, yielding a 200 bp wild-type DNA fragment and a 300 bp floxed DNA fragment. Presence of the *Tnfr1* alleles was detected using a combination of the following PCR primers: 5′-CTCTCTTGTGATCAGCACTG-3′, 5′-CTGGAAGTGTGTCTCAC-3′, 5′-CTGGAAGTGTGTCTCAC-3′ and 5′-CCAAGCGAAACATCGCATCGAGCGA-3′, yielding a 1400 bp wild-type DNA fragment and a 950 bp knockout DNA fragment.

### Generation of mixed bone marrow chimeras

CD45.1 recipient mice received a sublethal irradiation dose (800 cGy). At least 4 h later, the recipient mice were injected intravenously with a 1:1 mixture of BM cells (4 × 10^6^ cells in total) derived from CD45.1/2 and either *Ripk1*^FL/FL^ or *Ripk1*^ΔCD4^ (both CD45.2) donors. Validation of the reconstitution was performed 8 weeks later by collection of tail vein blood. Osmotic lysis of red blood cells was performed using ACK lysis buffer (10548E, Lonza, Basel, Switzerland) and lymphocytes were analyzed by flow cytometry. 12 weeks after reconstitution of the bone marrow, mice were euthanized and ratios of CD45.1/2 and CD45.2T cell numbers in spleen, mLN, and blood were analyzed by flow cytometry.

### Sample processing for flow cytometry and FACS

Thymi, spleens, and lymph nodes were harvested and stored in ice-cold PBS containing 3% heat-inactivated fetal calf serum (FCS) and 2 mM EDTA (15575020, Invitrogen, Waltham, MA, USA). Tissues were smashed on top of a 70 µm cell strainer using the plunger of a 3-ml syringe and single cells were subsequently collected by centrifugation at 500 × *g* and 4 °C during 5 min. For purification of splenocytes, an additional osmotic lysis of red blood cells was performed using ACK lysis buffer (10548E, Lonza).

### Flow cytometry

Single-cell suspensions were obtained as described, Fc-receptors were blocked with anti-CD16/CD32 antibodies (clone 2.4G2, BD Biosciences, Franklin Lakes, NJ, USA) and cells were stained with fluorochrome-conjugated monoclonal antibodies. The following antibodies were from BD Biosciences: BUV395-conjugated anti-CD3 (145-2C11), BV605-conjugated anti-CD4 (RM4-5), PE- and BUV395-conjugated anti-CD25 (PC61), V450-conjugated anti-CD44 (IM7), AF700-conjugated anti-CD45R/B220 (RA3-6B2), PE-conjugated anti-CD45.1 (A20), AF700- and PE-conjugated anti-CD62L (MEL-14), BUV737-conjugated anti-CD127 (SB-199), AF488-conjugated anti-FoxP3 (MF23), PerCP-Cy5.5-conjugated Ki-67 (B56) and FITC- and PE-CF594-conjugated anti-TCRγδ (GL3). The following antibodies were from Biolegend: APC-conjugated anti-CD3 (145-2C11), BV785-conjugated anti-CD4 (GK1.5), FITC- and PerCP-Cy5.5- and BV510-conjugated anti-CD8β (YTS156.7.7), BV785-conjugated anti-CD11b (M1/70), BV785-conjugated anti-CD11c (N418), BV785-conjugated anti-CD19 (6D5), BV421-conjugated anti-CD45R/B220 (RA3-6B2), PerCP-Cy5.5-conjugated anti-CD45.2 (104), BV421-conjugated anti-CD127 (A7R34), PE-conjugated anti-Bcl-2 (BCL/10C4), BV785-conjugated anti-F4/80 (BM8), BV785-conjugated anti-Ly-6G (1A8), APC-Cy7- and BV711-conjugated anti-TCRβ (H57-597) and BV785-conjugated anti-Ter-119 (TER-119). The following antibodies were from eBioscience (Waltham, MA, USA): eFluor450-conjugated anti-CD8α (53-6.7), PE-Cy5-conjugated anti-CD11b (M1/70), AF700- and PE-Cy5-conjugated anti-CD19 (1D3), PE-Cy7-conjugated anti-CD44 (IM7), FITC-conjugated anti-CD62L (MEL-14), APC-conjugated anti-FoxP3 (FJK-16s), PE-Cy5-conjugated anti-Gr-1 (RB6-8C5), and PE-Cy5-conjugated anti-Ter-119 (TER-119). Staining for cell viability was done using Fixable Viability Dye eFluor™ 780 or eFluor^TM^ 506 (eBioscience). Intracellular stainings for FoxP3, Ki-67, and Bcl-2 were performed using the Foxp3/Transcription Factor Staining Buffer Set (00-5523-00, eBioscience). Flow cytometry was performed on an LSR-II HTS, LSRFortessa™ 5-lasers, or FACSymphony™ A5 cytometer (BD Biosciences) using FACSDiva™ software, and data were analyzed using FlowJo™ v10 software.

### Cell enrichment and FACS

Thymus, spleens, and lymph nodes (axillary, brachial, inguinal, and mesenteric) were collected and single-cell suspensions were prepared as described before. For isolation of CD4^+^ single-positive thymocytes from the thymus, thymocytes were subjected to an in-house optimized procedure using Magnisort^TM^ magnets (eBioscience) for negative selection with Magnisort^TM^ streptavidin negative selection beads (MSNB-6002-74, eBioscience) and a biotinylated CD8α antibody (clone 53-6.7, eBioscience). For isolation of naive and CD44^high^ T cells from spleen and mLN, first CD4^+^ and CD8^+^ T cells were enriched with the Magnisort^TM^ Mouse T cell enrichment kit (8804-6820-74, eBioscience), according to the manufacturer’s instructions, or using Magnisort^TM^ magnets (eBioscience) for negative selection with Magnisort^TM^ streptavidin negative selection beads and biotinylated antibodies. The following antibodies were from eBioscience: anti-B220 (RA3-6B2), anti-Ly-6G/Ly-6C (RB6-8C5), anti-TER-119 (TER-119), anti-TCRγδ (GL3), anti-CD49b (DX5), anti-CD19 (1D3) and anti-CD24 (M1/69). The following antibodies were from Biolegend: anti-CD11b (M1/70) and anti-MHC-II (M5/114.15.2). The enriched T cells were then stained as described and sorting was performed using FACSAria™ II and FACSAria™ III cell sorters (BD Biosciences). The following gating strategies were applied: total T cells (live, CD4^+^ or CD8^+^), naive T cells (live, CD4^+^ or CD8^+^, CD25^-^, CD62L^+^ CD44^low^) and CD44^high^ T cells (live, CD4^+^ or CD8^+^, CD62L^-^ CD44^high^).

### Cell culture and stimulations

For the TNF-induced cell death assay, FACS-sorted CD4^+^ naive T cells or purified single positive CD4^+^CD8^-^ thymocytes were pre-treated with 1 µg/ml cycloheximide (C7698, Sigma-Aldrich) during 30 min, and 1 × 10^5^ cells were incubated on round-bottom 96-well plates in T cell medium (RPMI supplemented with 10% FCS, 400 µM sodium-pyruvate, 50 µM 2-mercaptoethanol, 20 µM glutamine, 100 IU/ml penicillin, 0.1 mg/ml streptomycin and non-essential amino acids) supplemented with 10 ng/ml IL-2 (VIB protein core, Ghent, Belgium), 1 ng/ml IL-7 (217-17, Peprotech, Rocky Hill, NJ, USA) and 10 ng/ml of either mTNF or hTNF (VIB protein core, Ghent, Belgium) during 20 h at 37 °C and 5% CO_2_. Cells were collected, stained as described, and cell death was analyzed by flow cytometry. For survival assays with a titration of IL-7, 1 × 10^5^ FACS-sorted naive T cells were incubated on round-bottom 96-well plates in T cell medium (RPMI supplemented with 10% FCS, 400 μM sodium-pyruvate, 50 μM 2-mercaptoethanol, 20 μM glutamine, 100 IU/ml penicillin, 0.1 mg/ml streptomycin and non-essential amino acids) with 10 ng/ml IL-2 (Protein core, VIB) and treated with different concentrations of IL-7 (217-17, Peprotech), ranging from 10 pg/ml to 10 ng/ml, for 72 h at 37 °C and 5% CO_2_. Cells were collected, stained, as described previously, and cell death was analyzed by flow cytometry using Fixable Viability Dye e780 (65-0865-14, eBioscience, San Diego, CA). For TCR-induced proliferation assays, FACS-sorted naive T cells were stained with CellTrace^TM^ Violet (C34557, Invitrogen) according to the manufacturer’s instructions. 1 × 10^5^ CTV-stained naive T cells were incubated on round-bottom 96-well plates, pre-coated with 5 μg/ml anti-CD3ε antibodies (100302, Biolegend), in T cell medium supplemented with 10 ng/ml IL-2 (Protein core, VIB) and 1 μg/ml soluble anti-CD28 (553294, BD Biosciences) during 72 h at 37 °C and 5% CO_2_. Cells were collected and stained as described for analysis of proliferation (CellTrace Violet dilution) and cell death (Fixable Viability Dye) by flow cytometry, or otherwise lysates were prepared for western blot analysis.

For the intrinsic/extrinsic apoptosis assays, FACS-sorted CD4^+^ naive T cells or purified CD4^+^ thymocytes were pre-treated with 1 µg/ml cycloheximide (C7698, Sigma-Aldrich) and, when indicated, 2 µM BV6 (S7597, Selleckchem), 2 µM GSK’872 (S8465, Selleckchem) or 50 µM zVAD (ALX-260-020-M005, Enzo Lifescience (VWR)) during 30 min. 7 × 10^4^ cells were incubated on round-bottom 96-well plates in T cell medium (RPMI supplemented with 10% FCS, 400 µM sodium-pyruvate, 50 µM 2-mercaptoethanol, 20 µM glutamine, 100 IU/ml penicillin, 0.1 mg/ml streptomycin and non-essential amino acids) supplemented with 10 ng/ml IL-2 (VIB protein core, Ghent, Belgium), 1 ng/ml IL-7 (217-17, Peprotech, Rocky Hill, NJ, USA) and 10 ng/ml of either mTNF or hTNF (VIB protein core, Ghent, Belgium) or 1 µM ABT-737 (S1002, Selleckchem) during 20 h at 37 °C and 5% CO_2_. Cells were collected, stained as described and cell death was analyzed by flow cytometry. The number of biological replicates (*n*) for each experiment is reported in the figure legends.

### Western blotting

FACS-sorted T cells or T cells from TCR-induced proliferation assays were collected and lysed in 1× Laemmli buffer containing 50 mM Tris-HCl pH 6.8, 2% sodium dodecyl sulphate (SDS), and 10% glycerol. Lysates were boiled during 10 min at 95 °C, proteins were separated by SDS-PAGE and transferred to a nitrocellulose membrane. Immunoblotting was performed by overnight incubation at 4 °C using the following primary antibodies: anti-RIPK1 (#610459, 1:2000, BD Biosciences), anti-caspase-8 (MAB3429, 1:1000, Abnova, Taipei, Taiwan) and anti-actin (69100, 1:20,000, MP Biomedicals, Irvine, CA, USA) followed by a 1 h incubation at RT using the following secondary antibodies: HRP-linked anti-mouse IgG (NA931, 1:3000, GE Healthcare, Chicago, IL, USA) and HRP-linked anti-rat IgG (NA935, 1:3000, GE Healthcare). Full length western blots can be found in Supplemental Materials.

### Sample processing for single-cell RNA sequencing

Single cells were prepared from the mLNs as described in ’Sample processing for flow cytometry and FACS’. Half of the cells were used for enrichment of T cells using the MagniSort™ Mouse T cell Enrichment Kit (8804-6820-74, eBioscience) according to the manufacturer’s instructions. The other half of the single cell suspensions were not enriched. Cells were subsequently stained with fluorochrome-conjugated monoclonal antibodies recognizing CD4 (clone RM4-5, BD Biosciences) and CD8β (clone YTS156.7.7, Biolegend), DAPI for cell viability, TruStain FcX Block (101320, BioLegend), the mouse cell surface protein antibody panel containing 165 oligo-conjugated antibodies and 9 TotalSeq-A isotype controls (TotalSeq-A, BioLegend) and a unique TotalSeq-A cell hashing antibody (BioLegend). Sorting of live CD4^+^ and CD8^+^ T cells from the enriched cells and sorting of total live cells from the unenriched cells was performed using FACSAria™ II and FACSAria™ III cell sorters (BD Biosciences). This resulted in two suspension of sorted cells per mouse which were tagged with unique hashing antibodies and combined in a 1:1 ratio to ensure enough T cells would be available for sequencing as T cell numbers are significantly reduced in the mLN of *Ripk1*^ΔCD4^ mice.

### Single-cell RNA sequencing

Three biological replicates per sample were loaded (consisting of a 1:1 combination of total live cells and enriched T cells) were multiplexed per lane using TotalSeq-A cell hashing antibodies. In the final analysis, only two of the three *Ripk1*^FL/FL^*Casp8* ^FL/FL^ samples had sufficient cells to analyse. Sorted single-cell suspensions at an estimated final concentration of 1500 cells/µl were loaded on a Chromium GemCode Single Cell Instrument (10x Genomics) to generate single-cell gel beads-in-emulsion (GEM). The scRNA-Seq libraries were prepared using the GemCode Single Cell 3′ Gel Bead and Library kit, version 3.1 (10x Genomics) according to the manufacturer’s instructions with the addition of amplification primers (3 nM each, 5′CCTTGGCACCCGAGAATT*C*C - 5′GTGACTGGAGTTCAGACGTGTGC*T*C) during cDNA amplification to enrich the TotalSeq-A cell surface and hashing protein oligos, respectively. Size selection with SPRIselect Reagent Kit (B23318, Beckman Coulter, Brea, CA, USA) was used to separate amplified cDNA molecules for 3′ gene expression and cell surface protein construction. TotalSeq-A protein library construction including sample index PCR using Illumina’s Truseq Small RNA primer sets and SPRIselect size selection was performed according to the manufacturer’s instructions. The cDNA content of pre-fragmentation and post-sample index PCR samples was analyzed using the 2100 BioAnalyzer (Agilent, Santa Clara, CA, USA). Sequencing libraries were loaded on an Illumina NovaSeq flow cell at VIB Nucleomics core with sequencing settings according to the recommendations of 10× Genomics, pooled in a 70:20:10 ratio for the combined 3’ gene expression, cell surface, and hashing protein samples, respectively. The Cell Ranger pipeline (10x Genomics, version 5.0.0) was used to perform sample demultiplexing and to generate FASTQ files for read 1, read 2, and the i7 sample index for the gene expression, cell surface, and hashing protein libraries. Read 2 of the gene expression libraries was mapped to the mouse reference genome (GRCm38.99). Subsequent barcode processing, unique molecular identifiers filtering, and gene counting was performed using the Cell Ranger suite. The average of the mean reads per cell across all gene expression libraries was 33,800, with an average sequencing saturation of 62%, as calculated by Cell Ranger. In total, 4 individual single-cell libraries were created in this study, entailing 56,740 cells.

### scRNA-seq data analysis

Separate samples were merged and the aggregate was processed via the following functions of the Seurat [42] (v 4.0.2) pipeline with default parameters unless specified otherwise: NormalizeData, FindVariableFeatures, ScaleData, RunPCA (npcs = 150), FindNeighbours (dims = 1:50), FindClusters (resolution = 1.3), RunTSNE (dims = 1:50), RunUMAP (dims = 1:50). Clusters were further curated manually based on marker gene expression. Differentially expressed genes were found using the FindMarkers function (adjusted *p-value* < 0.05). Figures were made using the DotPlot and DimPlot functions. Heatmaps were made using an inhouse built function based on the pheatmap package (v 1.0.12). To create the PCA figure, gene expression was averaged per sample using the AverageExpression function of Seurat and used as input for the prcomp function. Volcano plots were made in Graphpad Prism.

### Statistical analysis

Differences between experimental groups in all datasets were analyzed on absolute values or on Log2-transformed data (when the F-test showed differences between conditions) and is specified in the figure legend, comprising of unpaired t-tests, Fisher’s LSD one-way ANOVA or Fisher’s LSD two-way ANOVA. Results are expressed as mean ± sem. Statistical analyses were carried out using GraphPad Prism version 9.4.1 (Graphpad Software Inc., La Jolla, CA).

### Supplementary information

Legends of Suppl Figures and Tables

Suppl Figure S1

Suppl Figure S2

Suppl Figure S3

Suppl Figure S4

Suppl Figure S5

Suppl Table S1

Suppl Table S2

Suppl Table S3

Original Data File (Raw data)

Original Data File (Western blots)

## Data Availability

The single-cell sequencing has been deposited in the Gene Expression Omnibus under the ID GSE260973. All data needed to obtain the conclusions in this work are present in the paper or the Supplementary Materials figures or tables. All mouse lines, reagents, and software used are listed in the paper or the Supplementary Materials.
