# Supplementary material for: RIPK1 protects naive and regulatory T cells from TNFR1-induced apoptosis
Source: Cell Death Differ. 2024 May 11;31(6):820–32. doi: 10.1038/s41418-024-01301-w (PMC11164875; doi:10.1038/s41418-024-01301-w)
Supplement: Supplementary file 1 — Legends of Suppl Figures and Tables [file 41418_2024_1301_MOESM1_ESM.docx]

**Supplementary Figure Legends**

Figure S1: RIPK1-deficiency in conventional T cells results in a vast loss of peripheral naïve T cells and FoxP3^+^ Tregs

**(A)** Total live cells in the thymus of *Ripk1*^ΔCD4^ mice and *Ripk1*^FL/FL^ littermates from (*n* = 11) *Ripk1*^ΔCD4^ mice and (*n* = 13) *Ripk1*^FL/FL^ littermates. **(B)** Frequencies of total CD4^+^ (left) and CD8^+^ (right) peripheral T cells within the live cell population in the spleen and mLN of *Ripk1*^ΔCD4^ mice and *Ripk1*^FL/FL^ littermates combined from three independent experiments with (*n* = 7) *Ripk1*^ΔCD4^ mice and (*n* = 8) *Ripk1*^FL/FL^ littermates. **(C)** Stacked bar plots showing naïve (CD62L^+^CD44^low^), effector (CD62L^-^CD44^high^) and central memory (CD62L^+^CD44^high^) T cells within CD4^+^ (left) and CD8^+^ (right) populations in the mLN of *Ripk1*^ΔCD4^ mice and *Ripk1*^FL/FL^ littermates combined from three independent experiments and shown for *n* = 11 mice per condition. **(D)** Body weight of 12 weeks old *Ripk1*^ΔCD4^ mice (*n* = 16) and *Ripk1*^FL/FL^ littermates (*n* = 13), and 36 weeks old *Ripk1*^ΔCD4^ mice (*n* = 14) and *Ripk1*^FL/FL^ littermates (*n* = 13) of both sexes, represented as the percentage of the initial body weight at weaning age (3 weeks). **(E)** Stacked bar plots showing naïve, effector and central memory T cells within CD4^+^ (left) and CD8^+^ (right) populations in the mLN of *Ripk1*^K45A^ mice and *Ripk1*^+/+^ littermates, measured by flow cytometry combined from two independent experiments with (*n* = 8) *Ripk1*^ΔK45A^ mice and (*n* = 7) *Ripk1*^+/+^ littermates. **(F)** Total numbers of FoxP3^+^ regulatory T cells (Tregs) in the thymus of *Ripk1*^ΔCD4^ mice and *Ripk1*^FL/FL^ littermates combined from two independent experiments with (*n* = 6) *Ripk1*^ΔCD4^ mice and (*n* = 8) *Ripk1*^FL/FL^ littermates. **(G and H)** Stacked bar plots showing naïve, effector and central memory T cells within CD4^+^ and CD8^+^ populations in the mLN (G) and spleen (H) combined from three independent experiments with (*n* = 7) *Ripk1*^ΔFoxP3^ mice and (*n* = 8) *Ripk1*^FL/FL^ littermates. (A-H) Flow cytometry data are shown as mean ± sem, and (A, B, D, F) each dot represents an individual mouse. **Statistics:** Statistical significance was calculated by (A, D and F) unpaired t-tests (two-sided), or (B) Fisher’s LSD two-way ANOVA on absolute values, or (C, E, G and H) Log_2_-transformed data. ns = non-significant, * p < 0.05, ** p < 0.01, *** p < 0.001, **** p < 0.0001.

Figure S2: Mixed bone marrow chimeras reveal a survival disadvantage for peripheral RIPK1-deficient T cells in the blood

Bar graph depicting ratios of CD45.2 to CD45.1/2 B cells, TCRγδ^+^ T cells, CD4^+^ and CD8^+^ T cells and FoxP3^+^ Tregs, retrieved from the blood of mixed BM chimeras and measured by flow cytometry. A ratio of 1 indicates that the specific cell subset was reconstituted equally by the bone marrow cells of both donors. Data are combined from two independent experiments with *n* = 6 mice per group. Data are shown as mean ± sem, and each dot representing an individual mouse. **Statistics:** Statistical significance was calculated by Fisher’s LSD two-way ANOVA on Log_2_-transformed data. ns = non-significant, **** p < 0.0001.

Figure S3: RIPK1 is essential for survival of naïve T cells in a caspase-8-dependent manner

**(A)** Total live cells in the thymus of *Ripk1*^ΔCD4^*Casp8*^ΔCD4^ mice and *Ripk1*^FL/FL^*Casp8*^FL/FL^ littermates, measured by flow cytometry. Data were combined from two independent experiments with *n* = 10 mice per group. **(B)** Stacked bar plots showing CD4^-^CD8^-^ DN, CD4^+^CD8^+^ DP, CD4^+^ SP and CD8^+^ SP cells in the thymus of *Ripk1*^ΔCD4^*Casp8*^ΔCD4^ mice and *Ripk1*^FL/FL^*Casp8*^FL/FL^ littermates and combined from two independent experiments and shown for *n* = 15 mice per group. **(C)** Frequencies of total CD4^+^ and CD8^+^ peripheral T cells within the live cell population in the spleen and mLN of *Ripk1*^ΔCD4^*Casp8*^ΔCD4^ mice and *Ripk1*^FL/FL^*Casp8*^FL/FL^ littermates and combined from two independent experiments and shown for *n* = 8 mice per group. **(D)** Stacked bar plots showing naïve, effector and central memory T cells within CD4^+^ and CD8^+^ populations in the mLN. Data are representative of two independent experiments with (*n* = 3) *Ripk1*^ΔCD4^ (KO) and (*n* = 4) *Ripk1*^ΔCD4^*Casp8*^ΔCD4^ (DKO) mice and (*n* = 4) for each of their respective *Ripk1*^FL/FL^ and *Ripk1*^FL/FL^*Casp8*^FL/FL^ littermates. Absolute values and results of the statistical test are indicated in **Table S1**. **(E)** Western blot analysis of protein levels of cleaved caspase-8 fragments p43 and P18 on naïve CD4^+^ T cells, purified by cell sorting from the spleen and mLN of *Ripk1^ΔCD4^* mice, *Ripk1^FL/FL^* littermates and *Ripk1^ΔCD4^Casp8^ΔCD4^* mice, pre-treated with cycloheximide and stimulated with either mTNF or hTNF for 20h before lysis. The following gating strategies were applied: DP (live, CD3^-^CD4^+^CD8^+^TCR^-^), CD4^+^ (live, Lin^-^CD3^+^CD4^+^CD8^-^TCRβ^+^) and CD8^+^ (live, Lin^-^CD3^+^CD4^-^CD8^+^TCRβ^+^). **(F)** Cells were isolated from the mLN of *Ripk1*^ΔCD4^, *Ripk1*^ΔCD4^*Casp8*^ΔCD4^ mice and their respective *Ripk1^FL/FL^ and Ripk1^FL/FL^Casp8^FL/FL^* littermates and analysed by scRNA-seq. Dot plot displaying expression of marker genes per cluster used for cluster annotation. Size of dots represents the fraction of cells expressing a particular marker and color intensity indicates mean-normalized scaled expression levels. (A-D) Flow cytometry data are shown as mean ± sem, and (A and C) each dot representing an individual mouse. **Statistics:** Statistical significance was calculated by (A) unpaired t-test (two-sided), or (C) two-way ANOVA on absolute values, or (B or D) Log_2_-transformed data. ns = non-significant.

Figure S4: RIPK1-deficient naïve T cells proliferate but die following activation due to caspase-8-dependent apoptosis

**(A-D)** Cells were isolated from the mLN of *Ripk1*^ΔCD4^, *Ripk1*^ΔCD4^*Casp8*^ΔCD4^ mice and their respective *Ripk1*^FL/FL^ and *Ripk1*^FL/FL^*Casp8*^FL/FL^ littermates and analysed by scRNA-seq. (A-C) Heatmaps showing average expression levels of *Cd74,* *Igkc and Cd19* for the individual mouse in all clusters of the four genotypes. (D) Principal component analysis (PCA) of the average gene expression in the indicated clusters with each dot representing an individual mouse. **(E)** Percentages (left) and absolute numbers (right) of Ki-67^+^ cells within the population of naïve CD8^+^ T cells in the mLN of (*n* = 7) *Ripk1*^ΔCD4^ mice and (*n* = 8) *Ripk1*^ΔCD4^*Casp8*^ΔCD4^ mice, compared with (*n* = 9) *Ripk1*^FL/FL^ and (*n* = 8) *Ripk1*^FL/FL^*Casp8*^FL/FL^ littermates. **(F)** Heatmap showing average expression of selected genes from CD4^+^ naïve T cells. **(G)** CD8^+^ naïve T cells were purified by cell sorting from the spleen and lymph nodes of *Ripk1*^ΔCD4^, their *Ripk1*^FL/FL^ littermates, and *Ripk1*^ΔCD4^*Casp8*^ΔCD4^ mice and stimulated for 72 hours with different concentrations of IL-7. Cell death was determined by flow cytometry and represented as the percentage of dead cells within the CD8^+^ T cell population and are representative of three independent experiments and shown as the average of one biological experiment. **(H and I)** CD8^+^ naïve T cells were purified from the spleen and lymph nodes of *Ripk1*^ΔCD4^ and *Ripk1*^ΔCD4^*Casp8*^ΔCD4^ mice, and their respective *Ripk1*^FL/FL^ and *Ripk1*^FL/FL^*Casp8*^FL/FL^ littermates by FACS sorting, labeled with CellTrace Violet and stimulated during 72 hours with anti-CD3ε and anti-CD28 antibodies in the presence of IL-2. The proliferation of CD8^+^ T cells is represented by the dilution of CellTrace Violet staining measured by flow cytometry. (I) Percentages of dead CD8^+^ T cells were determined in every independent experiment by flow cytometry after 72 hours of stimulation with anti-CD3ε and anti-CD28 antibodies. Data are representative of (H) two independent experiments, or combined from (I-right) *n* = 2 or (I-left) *n* = 5 independent biological experiments. Data are shown as mean ± sem, and (E) each dot representing a mouse or (I) the average of technical replicates in an independent experiment. **Statistics:** Statistical significance was calculated by (E left) two-way ANOVA on absolute values, or (E right) Log_2_-transformed data, or (I) unpaired t-tests (two-sided). ns = non-significant, ** p < 0.01, *** p < 0.001, **** p < 0.0001.

Figure S5: Naïve T cells and FoxP3^+^ Tregs require RIPK1 for protection from TNFR1-induced apoptosis

**(A and B)** *Ripk1*^ΔCD4^ mice and *Ripk1*^FL/FL^ littermates were treated with anti-TNF-α antibodies or isotype control every 3-4 days for two weeks. Total numbers of (A-left) CD4^+^ or (B-left) CD8^+^ T cells, and stacked bar plots divided into naïve (CD62L^+^CD44^low^), effector (CD62L^-^CD44^high^) and central memory (CD62L^+^CD44^high^) (A-right) CD4^+^ and (B-right) CD8^+^ T cells in the spleen. Data are combined from two independent experiments with *n* = 8 in the *Ripk1^FL/FL^* (Isotype) and *Ripk1^ΔCD4^* (α-TNF) groups and *n* = 9 mice in the *Ripk1^ΔCD4^* (Isotype) and *Ripk1^FL/FL^* (α-TNF) groups. **(C)** CD4^+^ single-positive (SP) thymocytes were isolated from the thymus of *Ripk1*^ΔCD4^ mice, *Ripk1*^FL/FL^ littermates and *Ripk1*^ΔCD4^*Casp8*^ΔCD4^ mice, pre-treated with cycloheximide and stimulated during 20h with murine TNF (mTNF), human TNF (hTNF) or vehicle (Ctrl). Cell death was determined by flow cytometry by fixable viability dye (FVD) staining, and represented as the percentage of dead cells within the CD4^+^ T cell population. Data are combined and shown for *n* = 2 independent biological experiments. **(D)** Stacked bar plots showing naïve, effector and central memory T cells within CD4^+^ (left) and CD8^+^ (right) populations in the spleen of *Ripk1*^ΔCD4^ and *Ripk1*^ΔCD4^TNFR1^-/-^ mice and their respective *Ripk1*^FL/FL^ and *Ripk1*^FL/FL^TNFR1^-/-^ littermates and combined from two independent experiments and shown for *n* = 6 mice per condition. Absolute values and results of the statistical test are indicated in **Table S2**. Data are shown as mean ± sem, and (A and B) each dot representing an individual mouse or (C) the average of technical replicates in an independent experiment. **Statistics:** Statistical significance was calculated by (D) Fisher’s LSD one-way ANOVA, or (A, B, and C) two-way ANOVA on Log_2_-transformed data. ns = non-significant, * p < 0.05, ** p < 0.01, *** p < 0.001, **** p < 0.0001.

**Supplementary Table Legends**

Table S1: Statistical overview Fig. 3B and Fig. S3D

Statistical overview of differences between genotypes in Fig. 3B and Fig. S3D.

Table S2: Statistical overview Fig. 5F and Fig. S5D

Statistical overview of differences between genotypes in Fig. 5F and Fig. S5D

Table S3: Flow cytometry antibodies

Overview of used flow cytometry antibodies

Raw data file

Overview of raw data used for figure generation and statistics

***Western blots***

Overview of the full length western blots used in the paper
