# Supplementary figures and images for: RIPK1 protects naive and regulatory T cells from TNFR1-induced apoptosis
Source: Cell Death Differ. 2024 May 11;31(6):820–32. doi: 10.1038/s41418-024-01301-w (PMC11164875; doi:10.1038/s41418-024-01301-w)

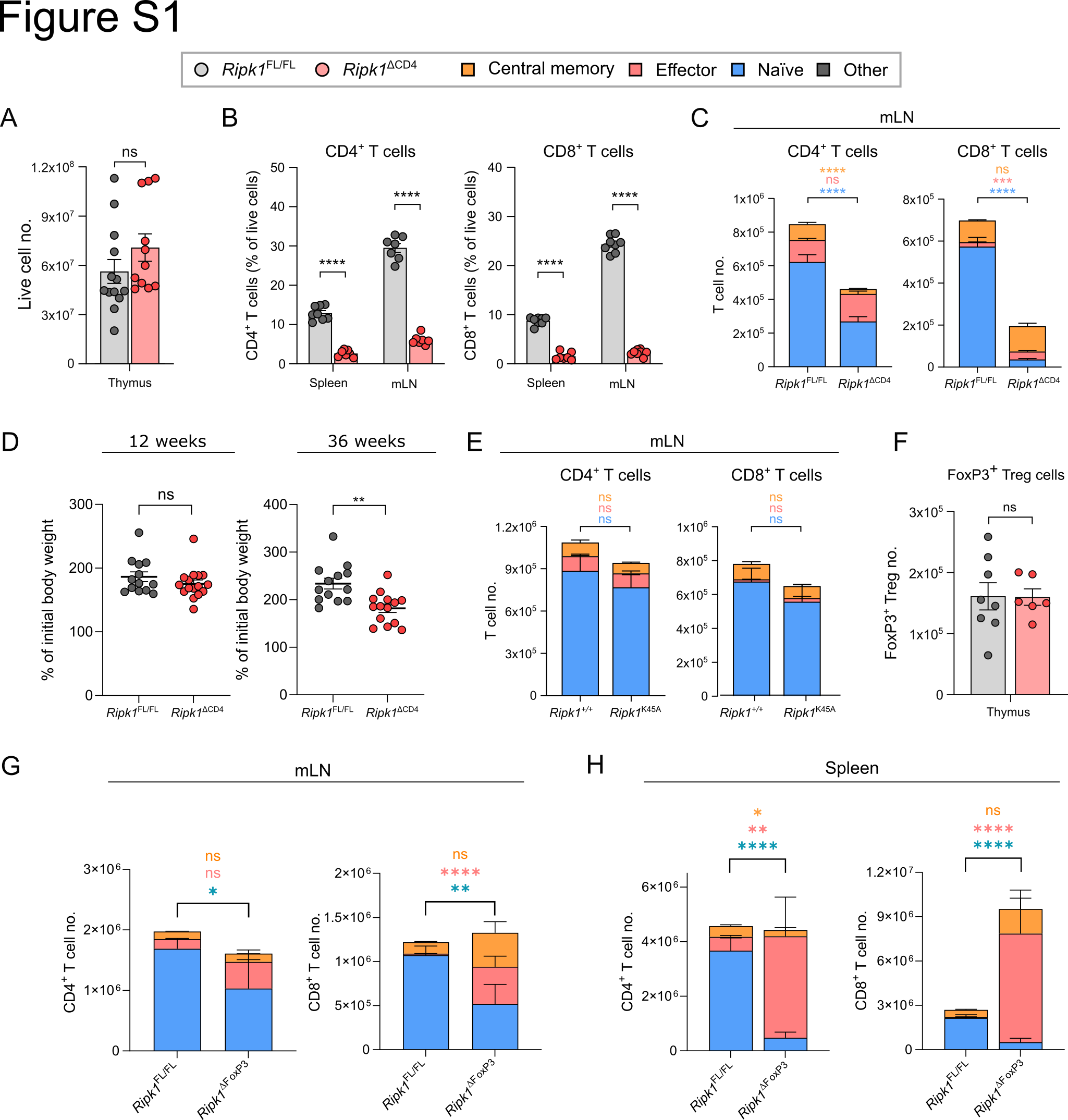

Supplement: Supplementary file 2 — Suppl Figure S1 [file 41418_2024_1301_MOESM2_ESM.png]

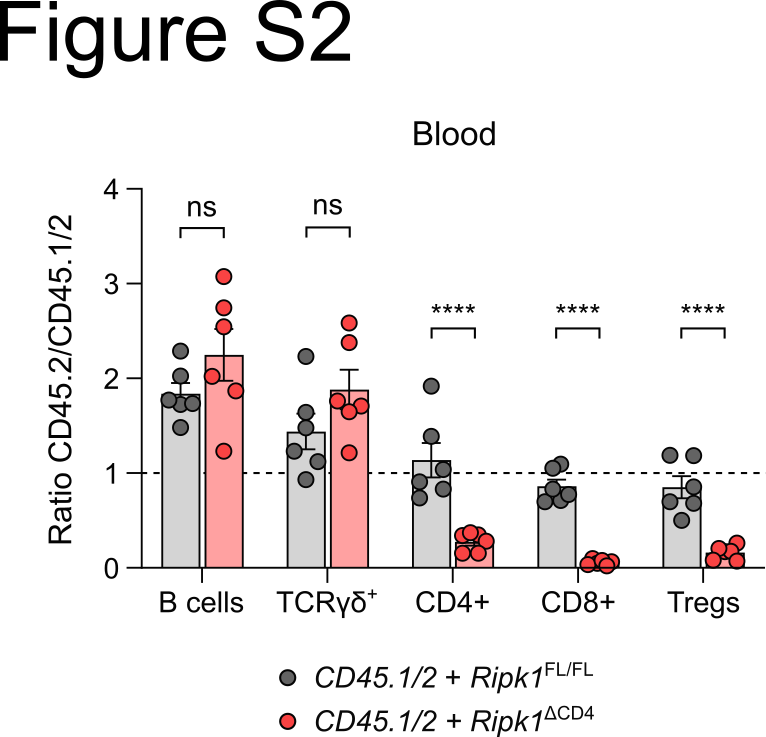

Supplement: Supplementary file 3 — Suppl Figure S2 [file 41418_2024_1301_MOESM3_ESM.png]

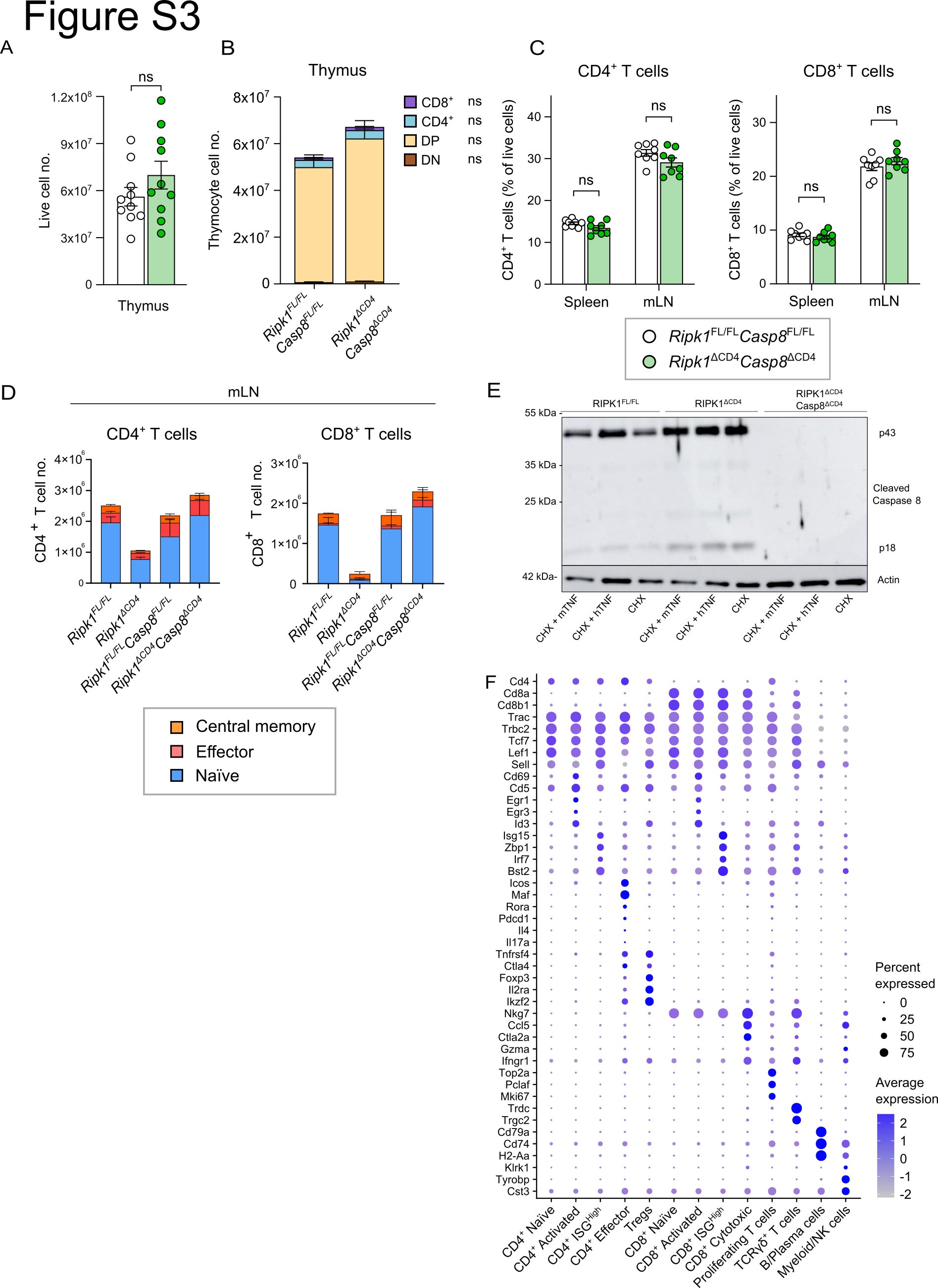

Supplement: Supplementary file 4 — Suppl Figure S3 [file 41418_2024_1301_MOESM4_ESM.png]

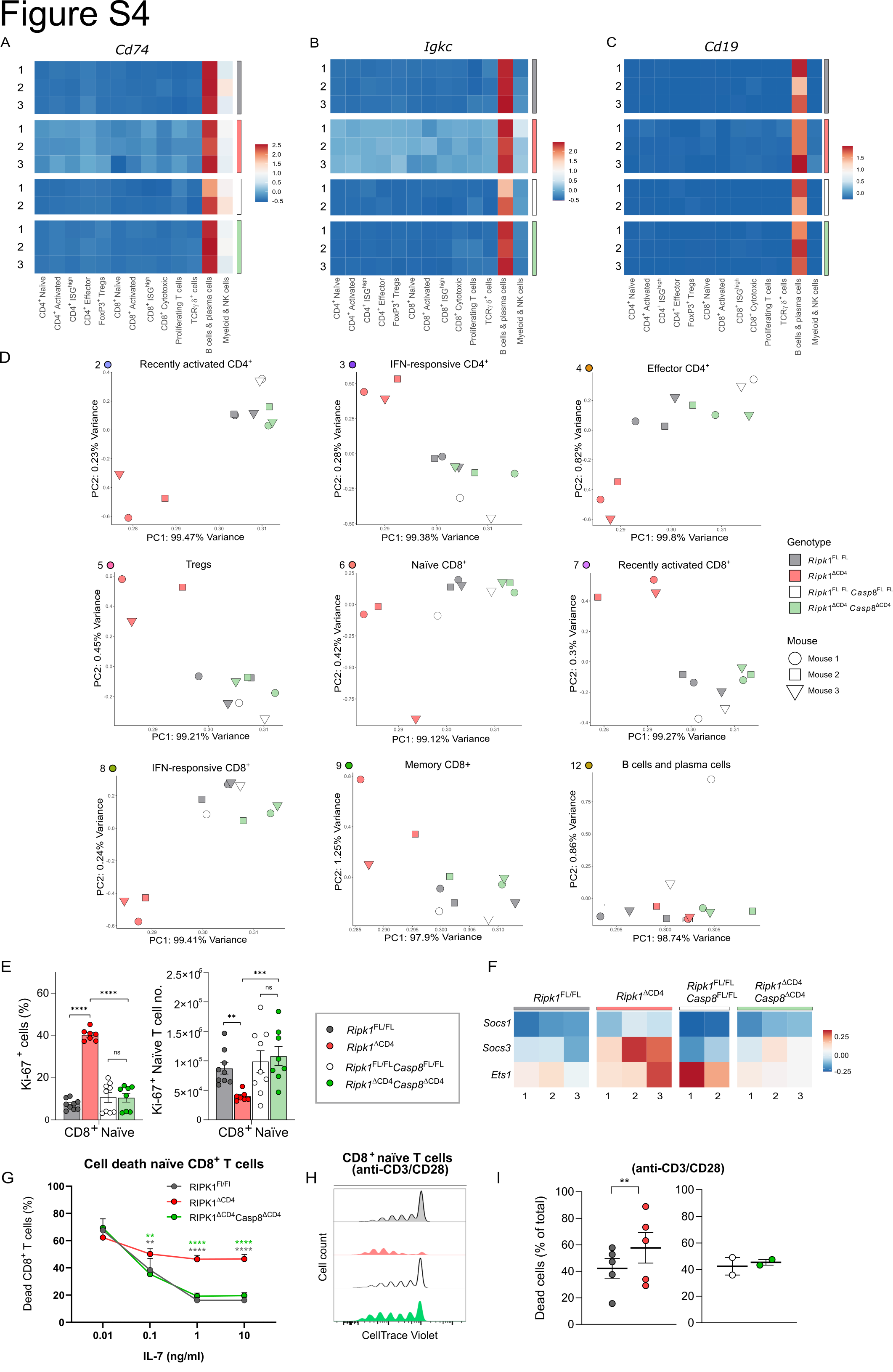

Supplement: Supplementary file 5 — Suppl Figure S4 [file 41418_2024_1301_MOESM5_ESM.png]

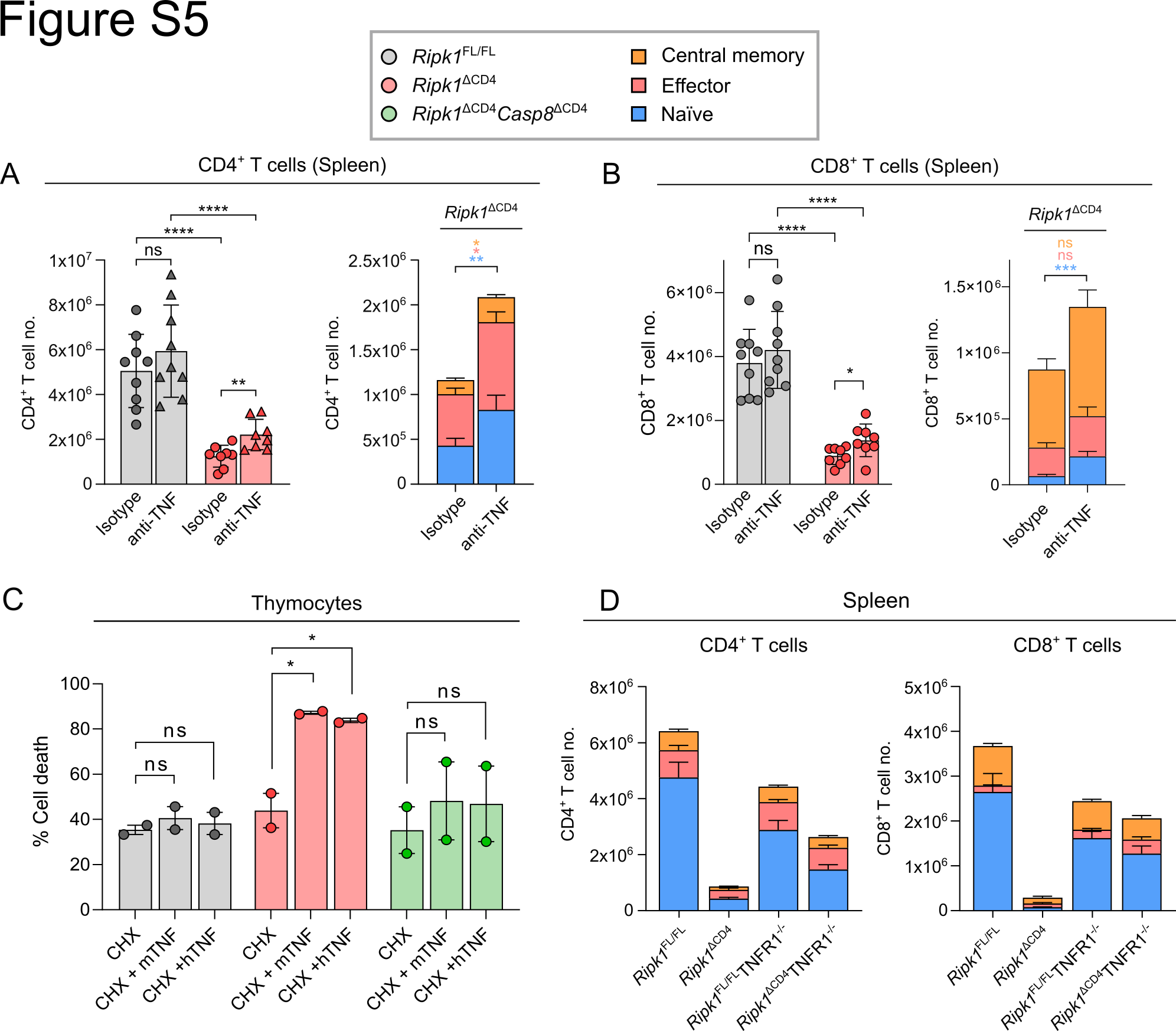

Supplement: Supplementary file 6 — Suppl Figure S5 [file 41418_2024_1301_MOESM6_ESM.png]

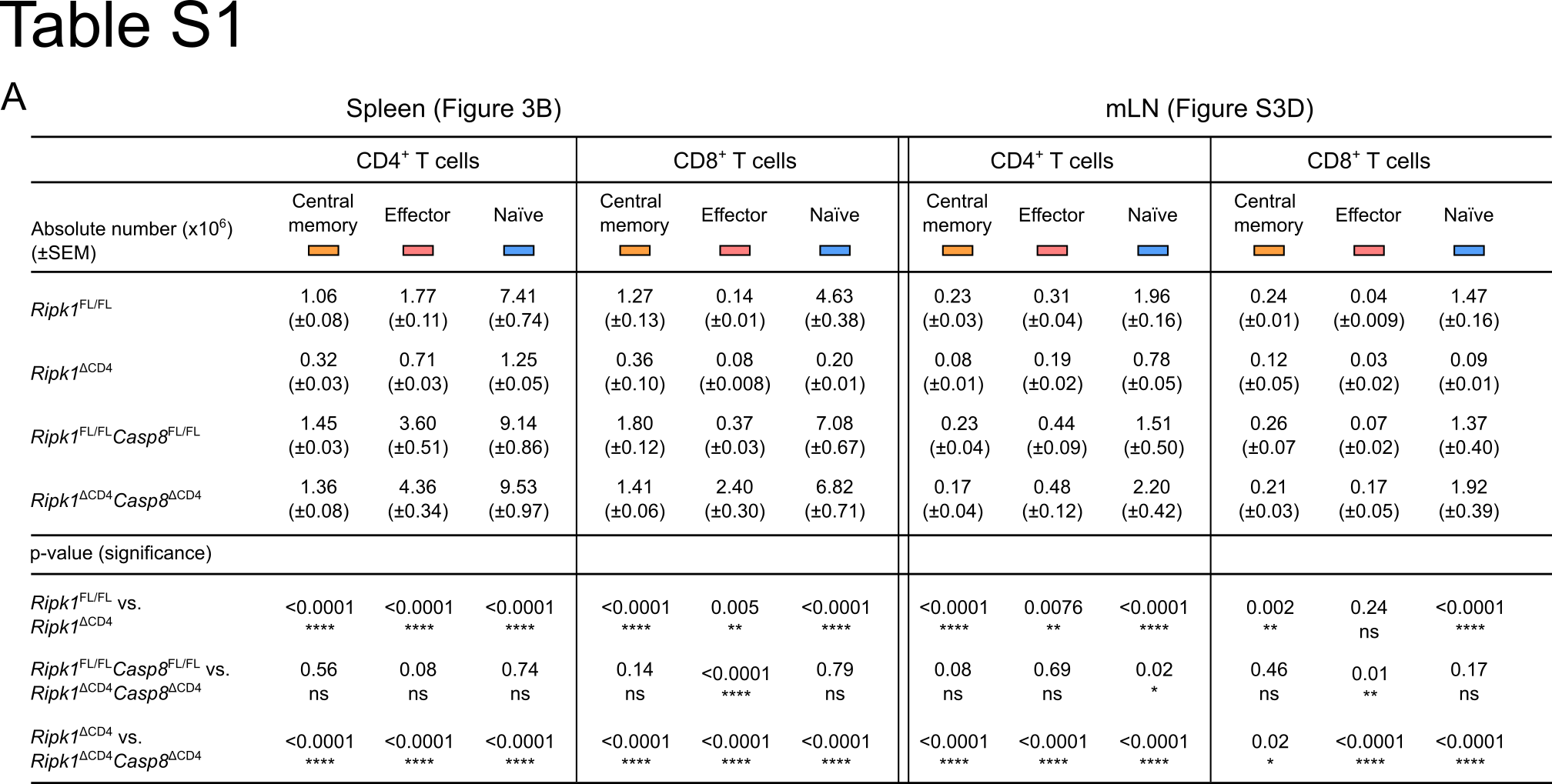

Supplement: Supplementary file 7 — Suppl Table S1 [file 41418_2024_1301_MOESM7_ESM.png]

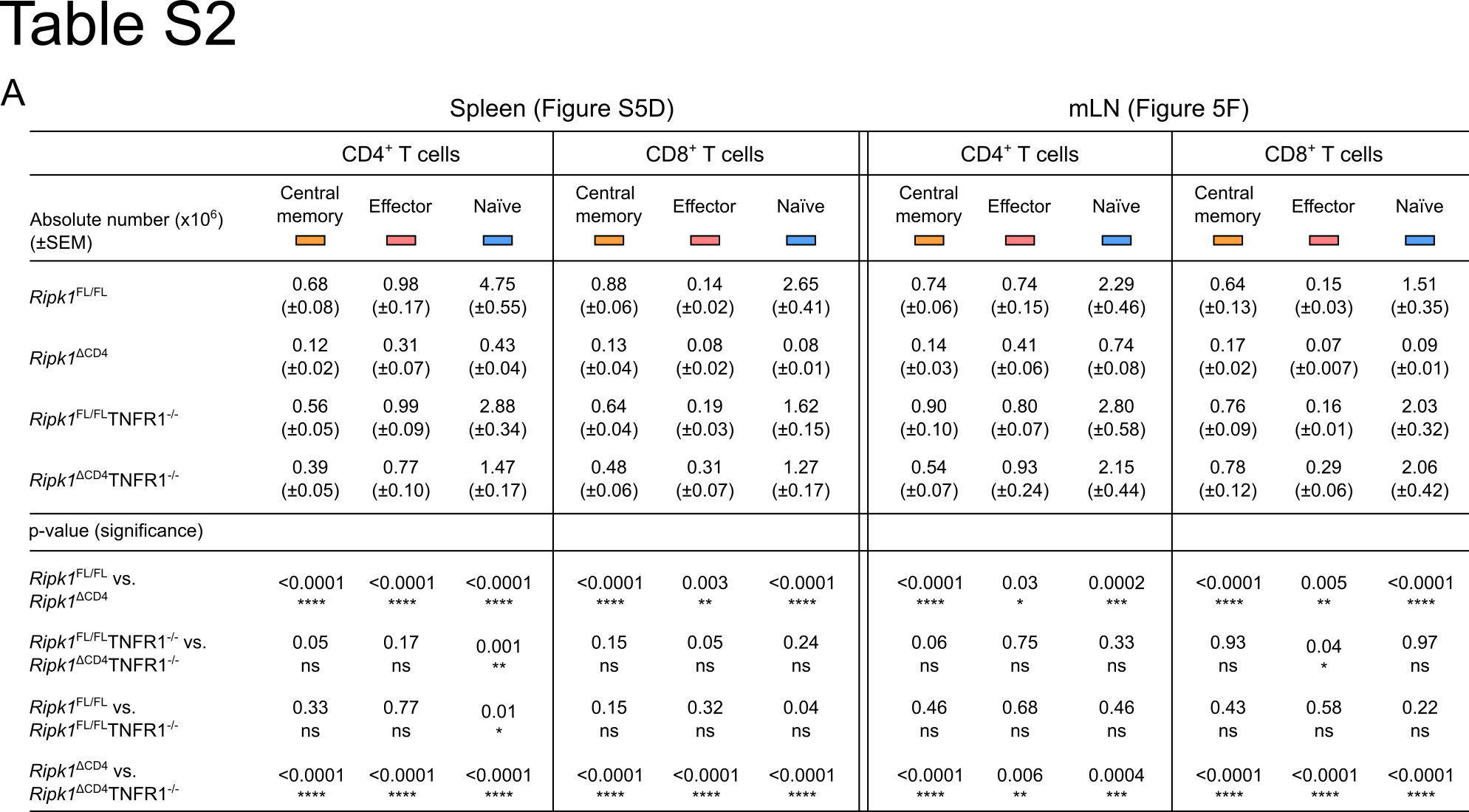

Supplement: Supplementary file 8 — Suppl Table S2 [file 41418_2024_1301_MOESM8_ESM.png]
