# Supplementary material for: RIPK1 protects naive and regulatory T cells from TNFR1-induced apoptosis
Source: Cell Death Differ. 2024 May 11;31(6):820–32. doi: 10.1038/s41418-024-01301-w (PMC11164875; doi:10.1038/s41418-024-01301-w)
Supplement: Supplementary file 11 — Original Data File (Western blots) [file 41418_2024_1301_MOESM11_ESM.docx]

**Full length western blots:**

**Figure 2A: Peripheral Naïve CD4^+^ and CD8^+^ T cells**


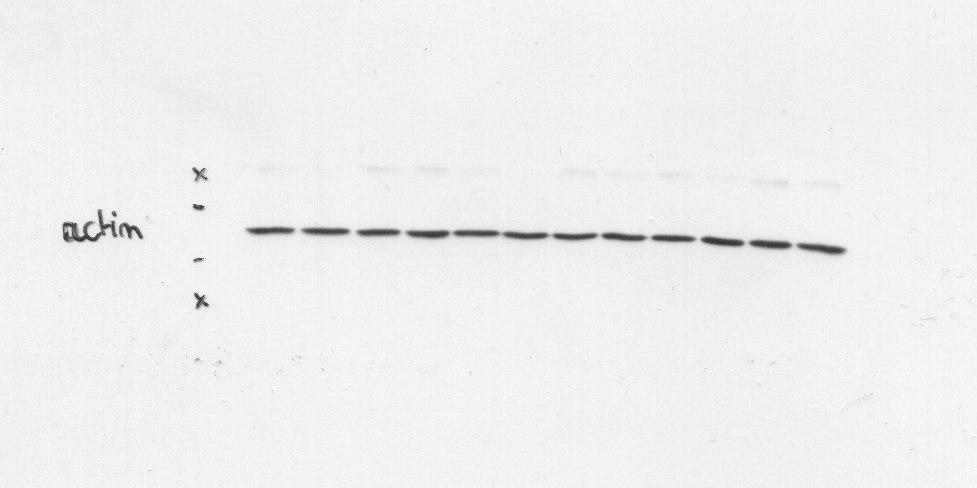

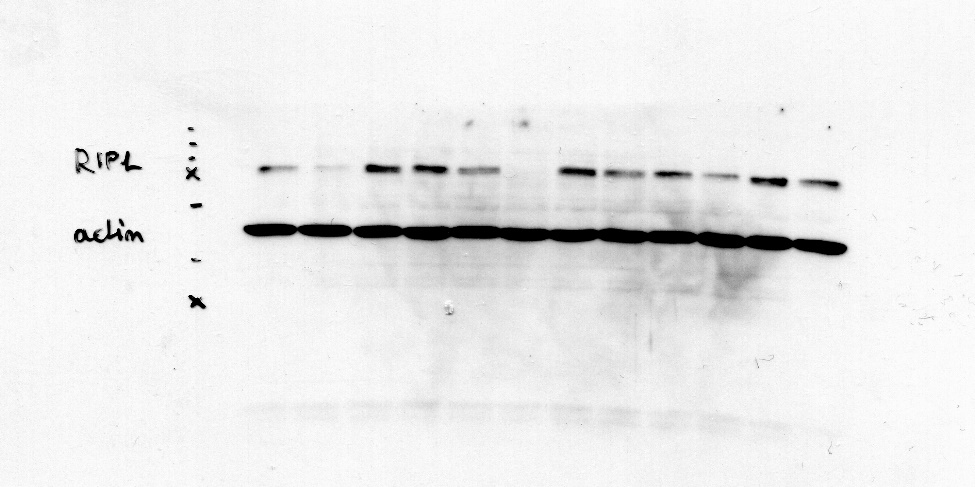


Actin

70

55

35

25

kDa

35

55

25

100

130

250

kDa

RIPK1

70

**Figure 2B: Naïve CD4^+^ and CD8^+^ T cells before and after anti-CD3/CD28 stimulation**


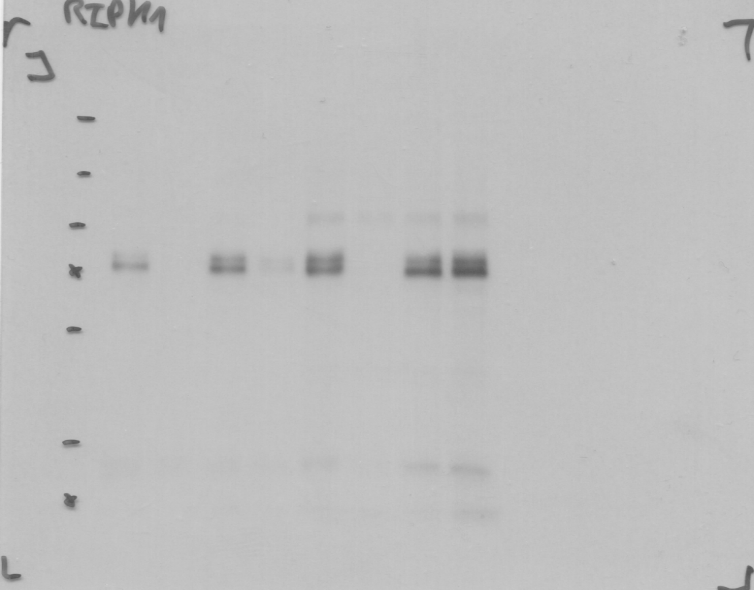


kDa

250

130

100

70

25

55

35

**Actin**


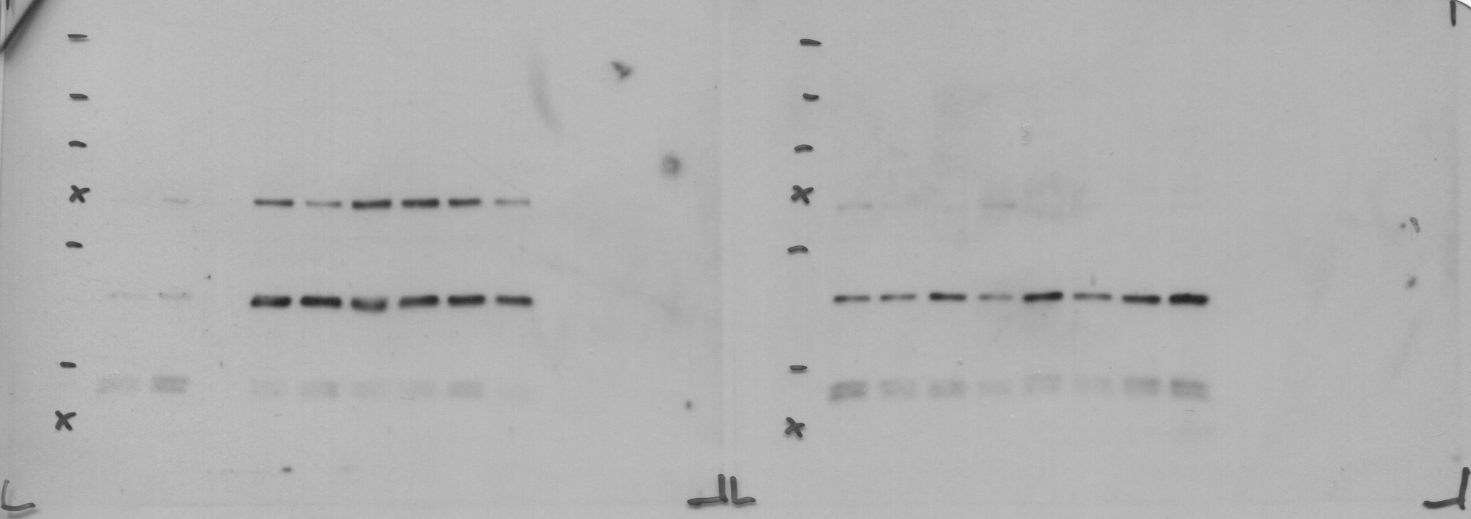


**RIPK1**

35

55

25

70

100

130

250

kDa

**Figure 3D: Thymus DP**


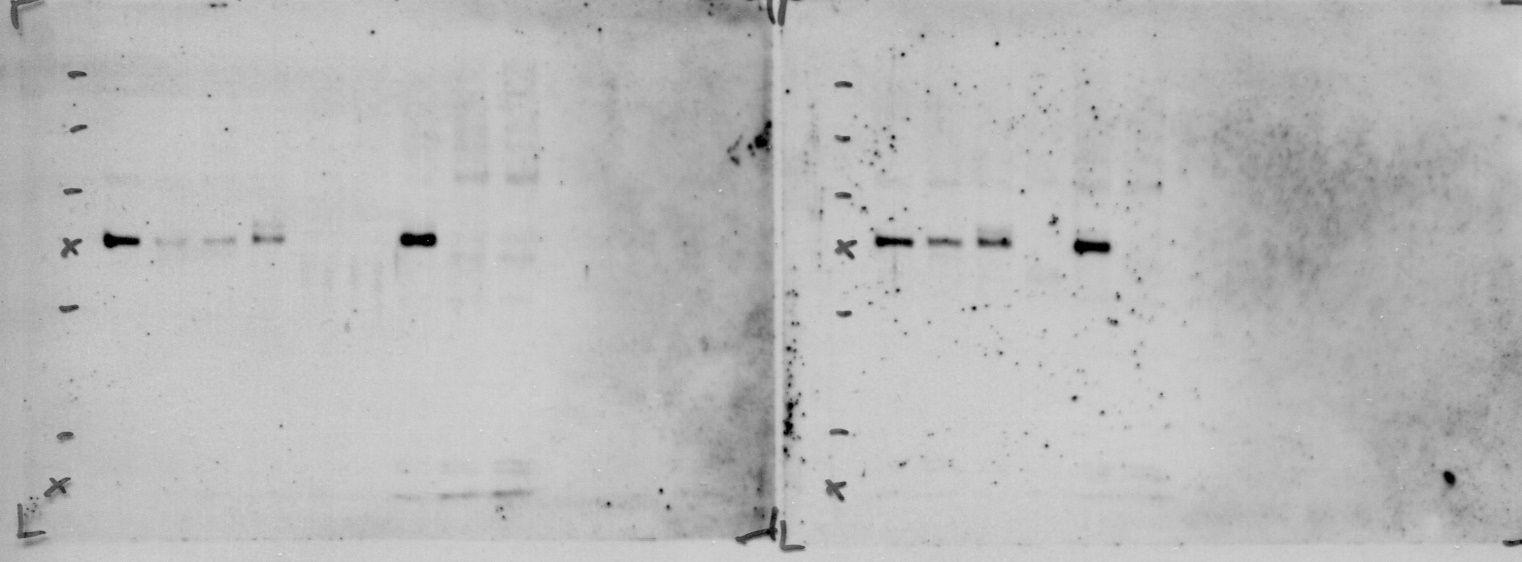


**RIPK1**

35

55

25

70

100

130

250

kDa


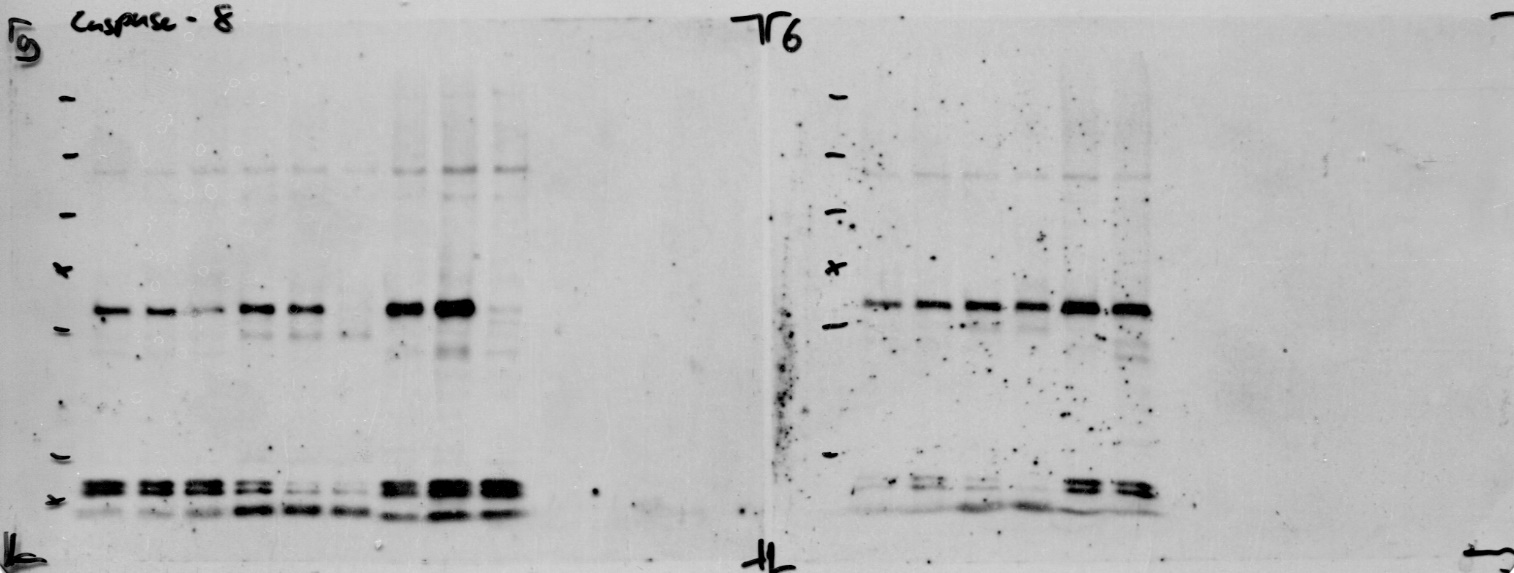


**Caspase-8**

35

55

25

70

100

130

250

kDa


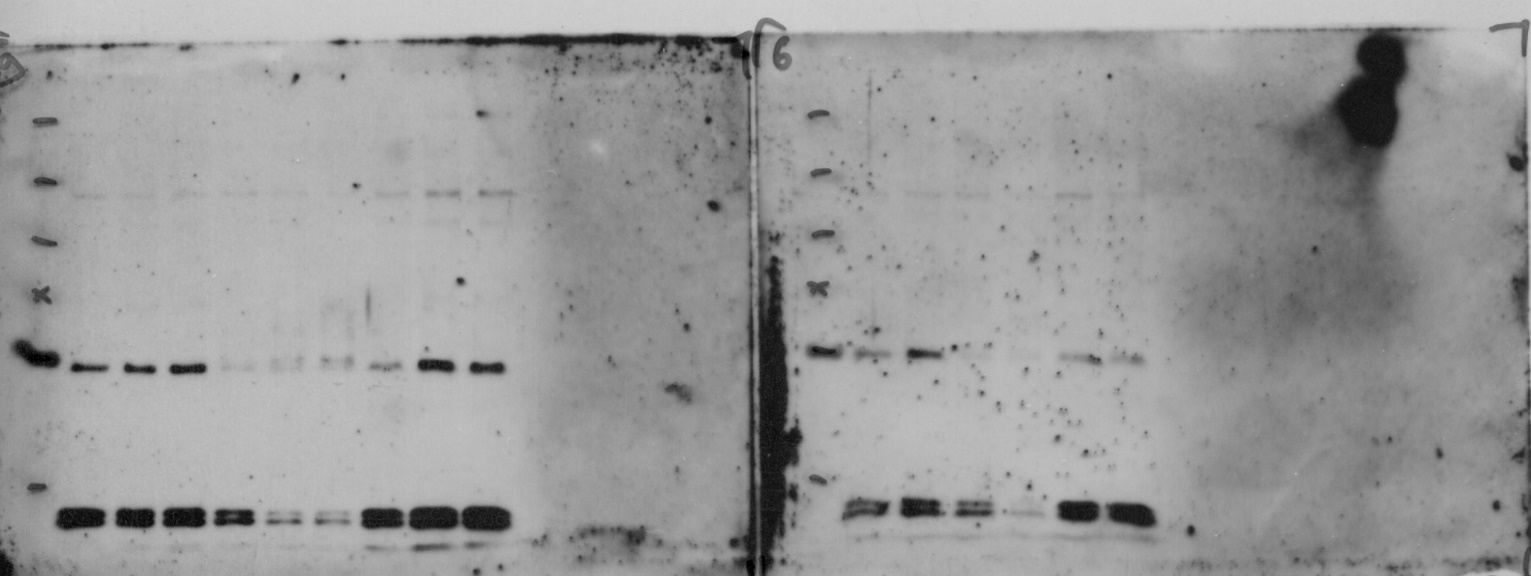


**Tubulin**

35

55

25

70

100

130

250

kDa

**Figure 3D: Thymus CD4-SP and CD8-SP**


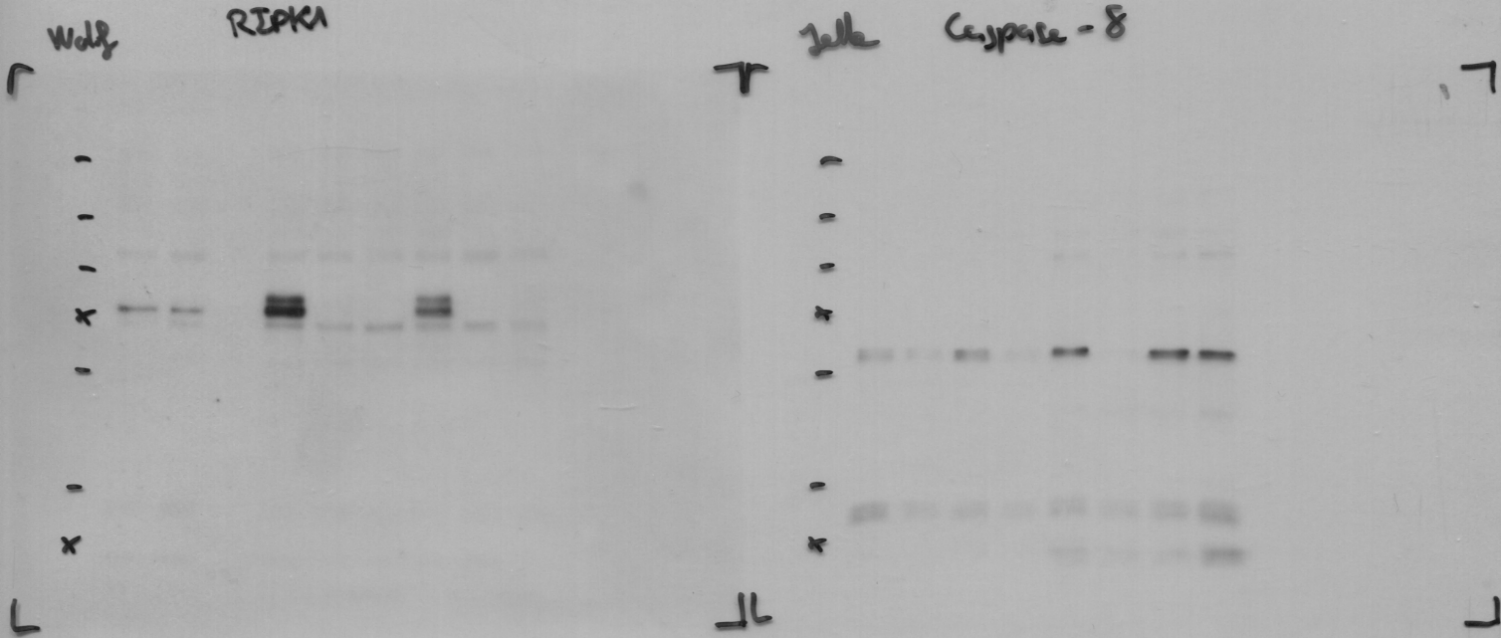


35

55

25

70

100

130

250

**RIPK1**


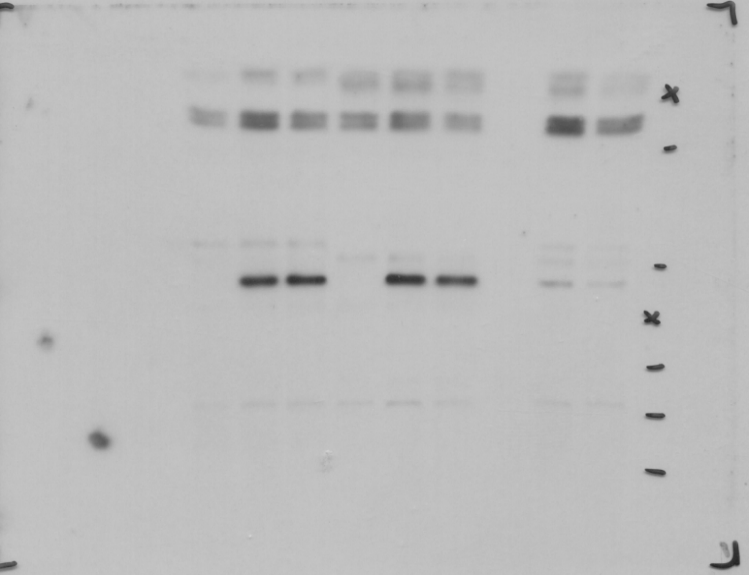


35

55

25

100

130

250

**Caspase-8**


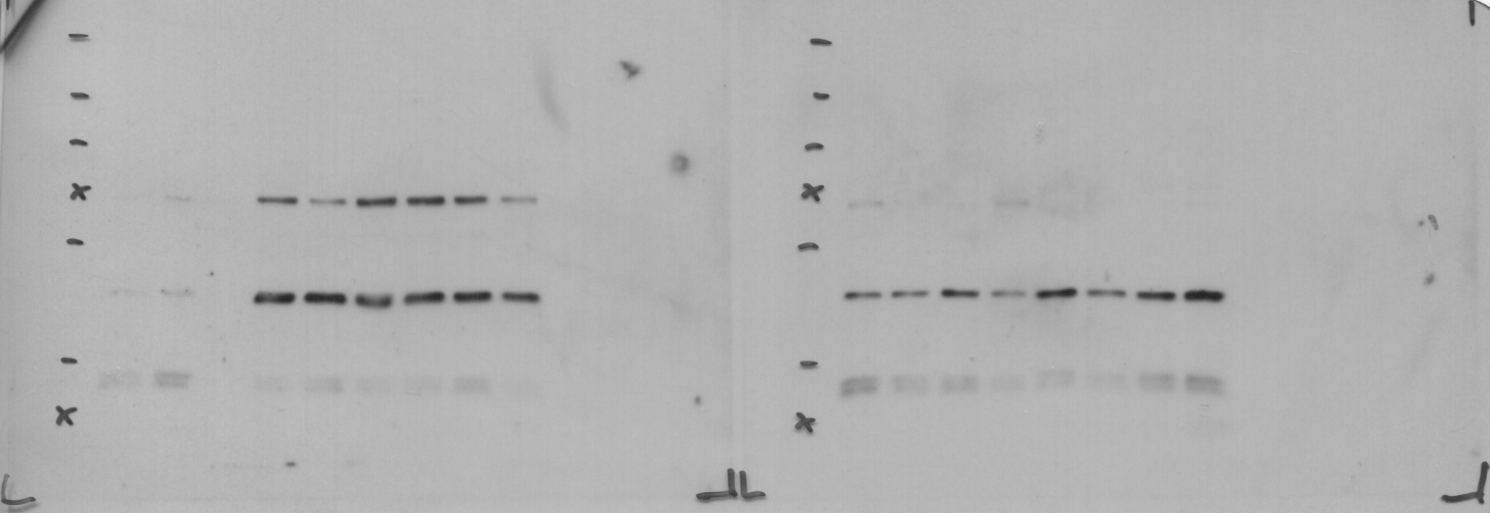


35

55

25

70

100

130

250

**Actin**

**Figure 3D: CD4 and CD8 T cells in the periphery**


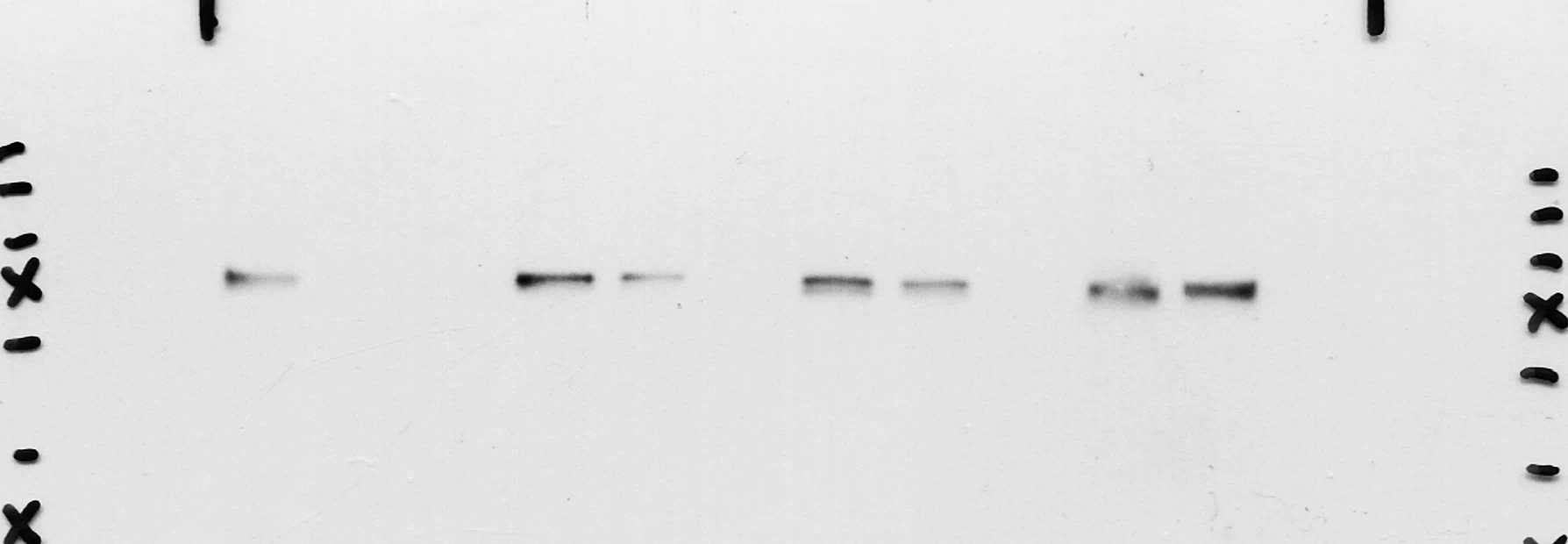


250

RIPK1

35

25

kDa

100

130

70

55


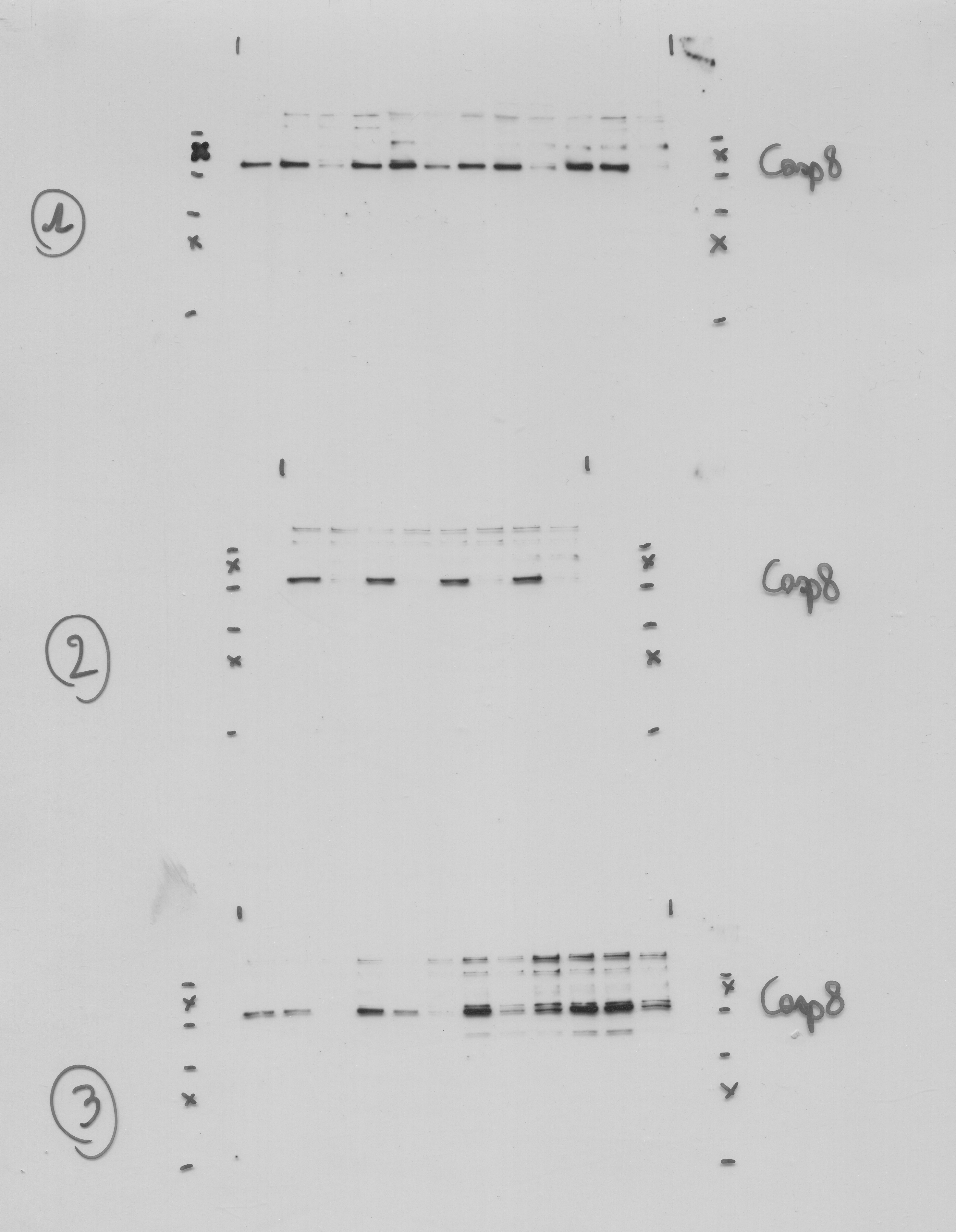


Caspase 8

35

55

25

70

100

kDa

15


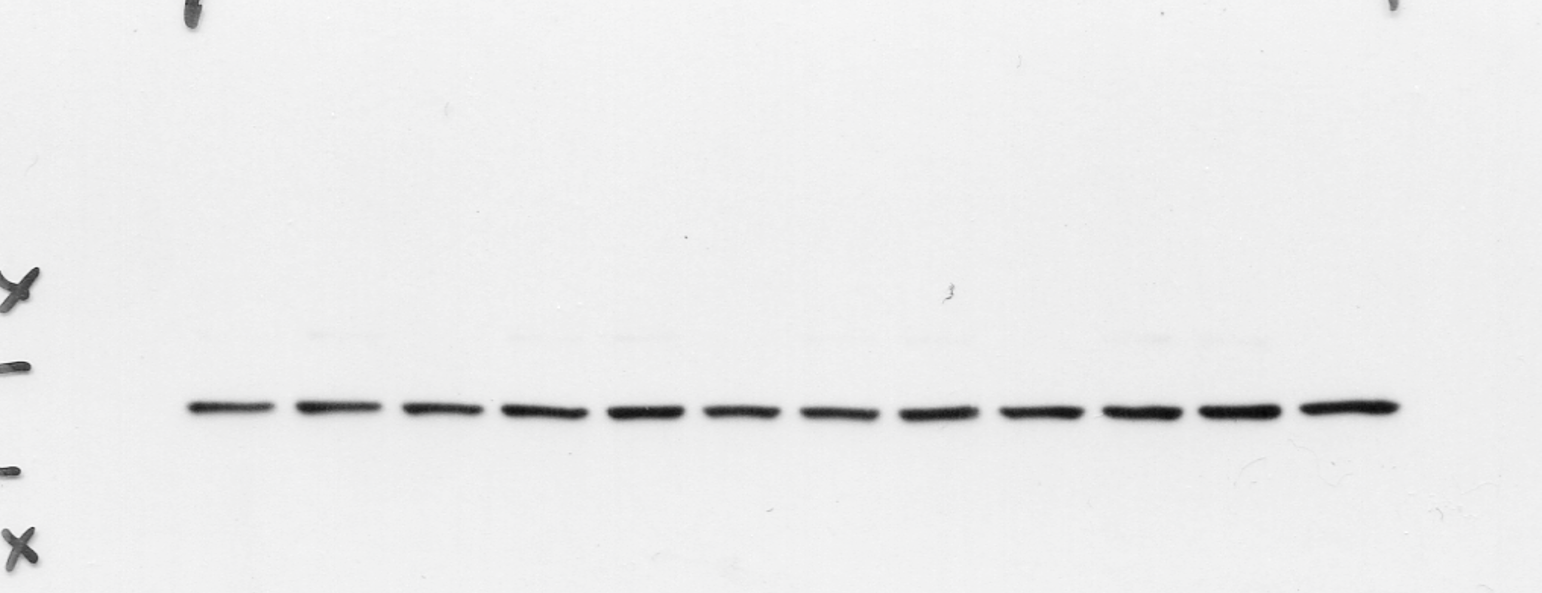


35

55

25

70

kDa

Actin

**Figure S3E: Cleaved caspase-8**


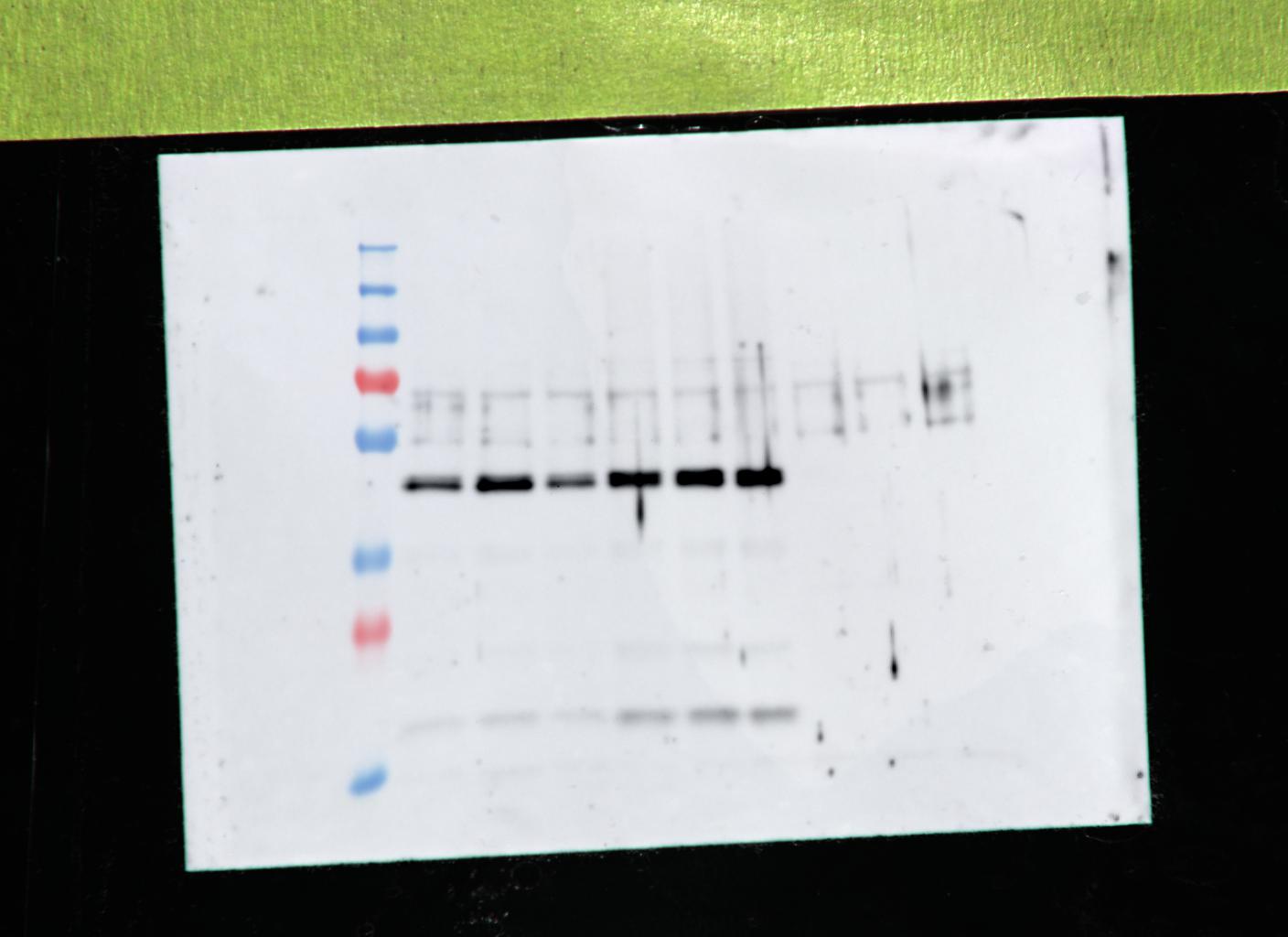


35

55

25

70

100

kDa

15

130

250

Cleaved
Caspase 8


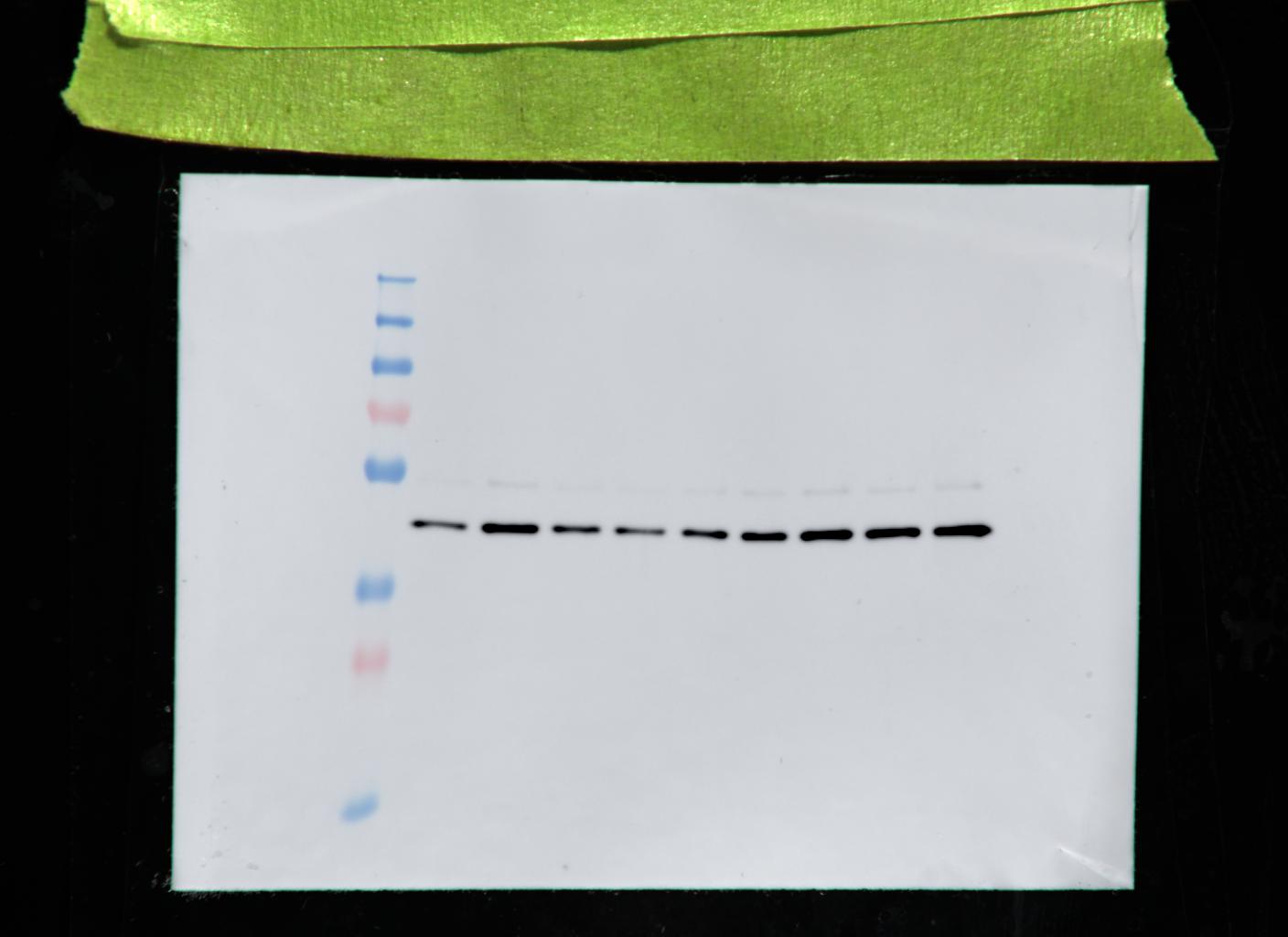


35

55

25

70

100

kDa

130

250

Actin
